# Jupiter microtubule‐associated homolog 1 (JPT1): A predictive and pharmacodynamic biomarker of metformin response in endometrial cancers

**DOI:** 10.1002/cam4.2729

**Published:** 2019-12-06

**Authors:** Nicholas W. Bateman, Pang‐Ning Teng, Erica Hope, Brian L. Hood, Julie Oliver, Wei Ao, Ming Zhou, Guisong Wang, Domenic Tommarello, Katlin Wilson, Tracy Litzy, Kelly A. Conrads, Chad A. Hamilton, Kathleen M. Darcy, Yovanni Casablanca, George Larry Maxwell, Victoria Bae‐Jump, Thomas P. Conrads

**Affiliations:** ^1^ Gynecologic Cancer Center of Excellence, Department of Obstetrics and Gynecology Uniformed Services University and Walter Reed National Military Medical Center Bethesda MD USA; ^2^ The John P. Murtha Cancer Center Uniformed Services University and Walter Reed National Military Medical Center Bethesda MD USA; ^3^ Henry M. Jackson Foundation for the Advancement of Military Medicine, Inc. Bethesda MD USA; ^4^ Department of Obstetrics and Gynecology Inova Fairfax Medical Campus Falls Church VA USA; ^5^ University of North Carolina at Chapel Hill Division of Gynecologic Oncology, Department of Obstetrics and Gynecology Chapel Hill NC USA

**Keywords:** endometrial cancer, hematological and neurological expressed 1, HN1, JPT1, Jupiter microtubule-associated homolog 1, metformin, proteomics

## Abstract

Preoperative use of metformin in obese women with endometrioid endometrial cancer (EEC) reduces tumor proliferation and inhibits the mammalian target of rapamycin pathway, though is only effective in select cases. This study sought to identify a predictive and/or pharmacodynamic proteomic signature of metformin response to tailor its pharmacologic use. Matched pre‐ and post‐metformin‐treated tumor tissues from a recently completed preoperative window trial of metformin in EEC patients (ClinicalTrials.gov: NCT01911247) were analyzed by mass spectrometry (MS)‐based proteomic and immunohistochemical analyses. Jupiter microtubule‐associated homolog 1 (JPT1) was significantly elevated in metformin responders (n = 13) vs nonresponders (n = 7), and found to decrease in abundance in metformin responders following treatment; observations that were verified by immunohistochemical staining for JPT1. Metformin response and loss of JPT1 were assessed in RL95‐2 and ACI‐181 endometrial cancer (EC) cell lines. We further identified that silencing of JPT1 abundance does not alter cellular response to metformin or basal cell proliferation, but that JPT1 abundance does decrease in response to metformin treatment in RL95‐2 and ACI‐181 EC cell lines. These data suggest that JPT1 represents a predictive and pharmacodynamic biomarker of metformin response that, if validated in larger patient populations, may enable preoperative EEC patient stratification to metformin treatment and the ability to monitor patient response.

## Graphical abstract

We report JPT1 (Jupiter microtubule‐associated homolog 1) as a predictive and pharmacodynamic biomarker candidate of metformin response in endometrial cancers.

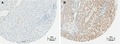

## 1 INTRODUCTION

Endometrial cancer (EC) is now the second most prevalent cancer among women in the USA and one of the few cancers for which disease incidence is on the rise, particularly for aggressive histologic subtypes.^1^, ^2^ Clinical management of EC includes total hysterectomy, bilateral salpingo‐oophorectomy, and pelvic and peri‐aortic lymph node dissection and can be followed by adjuvant treatment with chemotherapy and vaginal brachytherapy. Given the increasing incidence and the paucity of effective treatments for advanced and recurrent EC, novel therapeutic agents that could be used alone or in combination with more traditional hormonal and cytotoxic chemotherapy to combat these trends are under active investigation. Emerging therapeutics for EC include the antihyperglycemic drug, metformin.^3^, ^4^, ^5^ Owing to its effect of inhibiting hepatic gluconeogenesis, metformin is widely used clinically to manage type II diabetes. Metformin activates protein kinase AMP‐activated catalytic subunit alpha 2 (PRKAA2) that, in turn, stimulates a number of catabolic pathways including fatty acid oxidation, glycolysis, glucose uptake, and inhibits numerous ATP‐consuming processes, such as cholesterol and protein synthesis.^3^, ^6^ In the context of cancer, metformin has been shown to dramatically decrease proliferation of a number of different human cancer cell lines in vitro.^5^, ^7^, ^8^, ^9^ Metformin has also been shown to effectively repress tumor growth in xenograft models of breast, prostate, and colon cancer.^10^, ^11^, ^12^ In EC cells, metformin‐mediated PRKAA2 activation decreases cell growth by inhibiting mammalian target of rapamycin (mTOR) signaling.^5^ Epidemiologic studies of diabetic patients have revealed that those treated with metformin exhibit a significantly reduced risk to develop diverse cancers relative to non‐metformin‐treated patients.^13^, ^14^ Focused meta‐analyses have suggested that metformin improves disease outcome in diabetic cancer patients, as evidenced by a recent retrospective cohort study of 2529 diabetic patients with early stage breast cancer that found that women receiving metformin and adjuvant chemotherapy had a higher complete pathologic response rate than those not taking metformin.^15^ In EC, our team recently completed a preoperative window study investigating the impact of short‐term metformin treatment in women undergoing surgical staging for type I EC who were obese (BMI > 30) and/or have diabetes, but who were metformin treatment naïve (ClinicalTrials.gov: NCT01911247). We recently reported a comparative metabolomic analysis of serum and tumor tissues before and after metformin treatment stratified by response as measured by decreased abundance of the proliferation antigen MKI67 in tumor tissues by immunohistochemistry (IHC) analyses.^4^ Metformin responders exhibited significantly decreased levels of phospho‐PRKAA2 as well as several downstream mTOR targets in tumor tissues, and the serum metabolome reflected activated lipolysis suggesting the activation of fatty acid metabolism following metformin treatment.

In the current study, we set out to further investigate the matched pre‐ and post‐metformin‐treated endometrial tumor tissues with the goal of identifying protein‐based biomarkers of therapeutic response to metformin treatment in EC patients. We describe here a semiquantitative proteomics investigation of matched tumor tissues from the same cohort of pre‐ and post‐metformin‐treated patients collected during our preoperative study of metformin in obese EC patients and identification of Jupiter microtubule‐associated homolog 1 (JPT1) as a predictive and pharmacodynamic biomarker of metformin response in EC patients.

## 2 METHODS

### 2.1 Tissue specimens

Formalin‐fixed paraffin‐embedded (FFPE) tissues corresponded to matched pre‐ and post‐metformin treatment tumor biopsies for 20 obese EC patients participating in a preoperative window trial (ClinicalTrials.gov: NCT01911247) under IRB approved protocols focused on assessing the efficacy of metformin treatment in EC patients prior to surgical intervention recently conducted and described by our group.^4^ The clinical characteristics of our study cohort are identical to those described in Table 1 of our previous manuscript, that is, Schuler KM et al^4^ FFPE tissue fixation times were ≥6 hours. Eight micrometer tissue sections were cut by a microtome and placed on polyethylene naphthalate membrane slides. Aqueous H&E was used to stain the slides followed by laser microdissection (LMD) to harvest tumor cells from the sections in the sequence in which sections were generated. Laser microdissected tissues were collected in 45 µL of LC‐MS grade water (mean tumor cell area captured = ~16.1 ± 23 mm^2^).

**Table 1:** Top canonical signaling pathways or disease and biofunctions activated or inhibited among 79 proteins significantly altered between metformin responder vs nonresponder

Canonical pathways	*P*‐value	Proteins (#)
Glucocorticoid receptor signaling	2.75E−05	8
BER pathway	8.13E−04	2
AMPK signaling	1.05E−03	5
Mismatch repair in eukaryotes	1.45E−03	2
Aryl hydrocarbon receptor signaling	1.62E−03	4

Functional annotation	*P*‐value	Activation z‐score	Proteins (#)
Viral infection	1.18E−03	2.776	18
Cell proliferation of tumor cell lines	1.00E−06	2.702	25
Repair of DNA	1.04E−04	2.599	8
Migration of cells	7.75E−06	2.265	26
Proliferation of prostate cancer cell lines	1.63E−04	2.157	8
Death of embryo	2.87E−06	−1.937	7
Neural tube defect	3.74E−03	−1.949	4
Apoptosis	2.15E−07	−2.164	34
Cell death of central nervous system cells	2.39E−03	−2.173	6
Cell death	1.28E−10	−2.288	45

### 2.2 Liquid chromatography‐tandem mass spectrometry proteomics

Tissue samples were processed and analyzed by high‐resolution liquid chromatography‐tandem mass spectrometry (LC‐MS/MS) on an Orbitrap Velos MS (Thermo Fisher) and JPT1 IP samples (preparation described below) were similarly analyzed on a Fusion Lumos MS (Thermo Fisher) as previously described.^16^, ^17^ Peptide identifications were filtered to include peptide spectrum matches (PSMs) passing an FDR < 1.0%. Proteins included in subsequent quantitative analyses were required to have a minimum of two PSMs. Pathway analyses were performed using Ingenuity Pathway Analysis (Qiagen).

### 2.3 Immunohistochemistry

Immunohistochemistry (IHC) analyses were performed on a previously described^4^ tissue microarray constructed from pre‐ and post‐metformin‐treated, FFPE tumor biopsies. IHC was carried out in the Bond Autostainer (Leica Microsystems Inc). Bond Dewax solution (AR9222) was used to dewax the slides and Bond Wash solution (AR9590) was used to hydrate the slides. Bond‐Epitope Retrieval solution 1, pH‐6.0 (AR9961), was used for antigen retrieval at 100°C for 20 minutes. Primary antibodies were diluted in Bond Primary Antibody Diluent (Leica, catalog #AR9352) and incubated at room temperature. Slides were incubated with primary antibody rabbit polyclonal anti‐HN1 (Sigma‐Aldrich—HPA059729, Bromma, Sweden, 1:100) for 2 hours. Antibody detection was performed using the Bond Polymer Refine detection system (DS9800). Stained slides were then dehydrated and coverslips were placed on top. Positive controls included assessment of primary antibody performance in a tissue microarray (TMA) containing multiple human tissue types positive or negative for hematological and neurological expressed 1 (HN1) and a second TMA that contained human endometrial tissue. Negative controls included assessment of tissue controls without primary antibody to confirm that background signals were below detection thresholds.

### 2.4 IHC image analysis

Stained slides were digitally scanned at 20× magnification using Aperio ScanScope‐XT (Aperio Technologies). Digital images were stored and analyzed within an Aperio eSlideManager Database. TMA images were segmented into individual cores using the Tissue Studio TMA portal (Tissue Studio version 2.7 with Tissue Studio Library v4.4.2; Definiens Inc, Munich, Germany). Epithelial cell‐enriched regions were digitally separated out for analysis using Tissue Studio Composer software (Definiens). Tissue Studio's Nuclear Algorithm was then used to detect and enumerate cells that expressed JPT1. The percentage of positive nuclei and an H‐score (formula = (% at 1+) * 1 + (% at 2+) * 2 + (% at 3+) * 3) were determined for each TMA core.

### 2.5 Cell culture and metformin treatment

RL95‐2 was purchased from ATCC (Manassas, VA, CRL‐1671) and maintained in DMEM:F‐12 medium (ATCC 30‐2006) supplemented with 10% fetal bovine serum (ATCC 30‐2020), 100 U/mL penicillin/100 µg/mL streptomycin (ATCC ATCC 30‐2300), and 0.005 mg/mL insulin (Sigma‐Aldrich, I9278). ACI‐181 (RRID:CVCL_N828),^18^ a model of endometrioid endometrial cancer (EEC) was a gift from Dr John Risinger (Department of Obstetrics, Gynecology and Reproductive Biology, Michigan State University) and cultured in DMEM:F12 supplemented with 10% fetal bovine serum, 100 U/mL penicillin, and 100 µg/mL streptomycin. Both cell lines were maintained at 37°C and 5% CO_2_. Metformin was purchased from Tocris (2864). For the metformin time course experiments, 100 000 and 200 000 of ACI‐181 and RL95‐2 were seeded in 6‐well plates and treated with 20 mmol/L of metformin for 96 and 120 hours. Two independent experiments were performed with one biological sample for each treatment condition per cell line.

### 2.6 Cell proliferation assay

Cells were trypsinized using 0.25% trypsin‐EDTA (ATCC 30‐2101). Cells were counted with 0.4% Trypan Blue (Bio‐Rad 145‐0013) with an automated cell counter (TC10, Bio‐Rad). Viable cells were plated in 96‐well plates at 5000 cells per well for each cell lines. For the metformin dose‐response assay, media was removed 24 hours later and replaced with fresh media containing metformin (0, 1.56, 3.13, 6.25, 12.5, 25, 50, 100 mmol/L) followed by incubation for 72 hours. 3‐(4,5‐dimethylthiazol‐2‐yl)‐5‐(3‐carboxymethoxyphenyl)‐2‐(4‐sulfophenyl)‐2H‐tetrazolium (MTS) CellTiter 96 Aqueous One Solution Cell Proliferation Assay (Promega, G358B) was used to assess cell viability according to the manufacturer's instructions. Briefly, after 3 hours incubation at 37°C, a microplate spectrophotometer (xMark, Bio‐Rad) was used to measure the absorbance at 490 nm. Three independent experiments were performed for each cell line with three technical replicates per metformin dose. For the cell proliferation assay without metformin treatment, 3500 and 5000 cells were plated in 96‐well plates in triplicate. Two independent experiments were performed. Seventy‐two hours following transfection with siNT or siJPT1 siRNA, cell viability was assessed daily by MTS assay as described above each day for four consecutive days for both the RL95‐2 and ACI‐181 cell lines.

### 2.7 JPT1 knockdown by SIRNA

ON‐TARGETplus Human JPT1 (HN1) siRNA SMART pool (L‐021065‐02‐0005) and ON‐TARGETplus Human Non‐Targeting Control pool (D‐001810‐10‐05) were purchased from Dharmacon. TransIT‐siQUEST transfection reagent (Mirus Bio MIR2110) was used for the small interfering RNA (siRNA) transfections of the RL95‐2 and ACI‐181 cell lines. For both cell lines, cells were seeded in 6‐well plates at 200 000 per well and transfected after 24 hours. Cells were transfected with 50‐100 nmol/L siRNA according to the manufacturer's protocol. Seventy‐two hours posttransfection, cells were trypsinized, counted, and seeded for cell viability assay. Cells were also harvested at 72 and 168 hours after transfection to assess JPT1 knockdown by immunoblotting and qPCR analyses. Two biological replicates were performed for each cell line. To confirm knockdown of JPT1, we performed qPCR analyses of RL‐95‐2 cells transfected with JPT1 siRNA. Specifically, sub‐confluent RL‐95‐2 cells were washed with cold PBS (ATCC 30‐2200), lysed in TRIzol (Invitrogen 15596018), and total RNA was extracted according to the manufacturer's instructions followed by purification using RNeasy Mini Kit (Qiagen 74104). Superscript VILO cDNA synthesis kit (Invitrogen 11754‐250) was used to prepare cDNA from 250 ng of total RNA by reverse transcription. *JPT1* (Hs00602957_m1) and *GAPDH* (Hs99999905_m1) TAQMAN assays were obtained from Applied Biosystems (Thermo Fisher). Quantitative PCR was performed with TAQMAN gene expression master mix (Applied Biosystems 4304437) using 10 ng of total cDNA. The annealing temperature was 60°C for the TAQMAN reaction for 40 cycles (ABI GeneAmp 9700 DNA thermal cycler). Comparison of Delta‐Ct values for *JPT1* vs corresponding *GAPDH* Delta‐Ct values was performed. Two independent experiments were performed with triplicate technical replicates. For *JPT1*, the range of Ct was 23.96‐28.17 and for *GAPDH*, the range of Ct was 21.84‐23.74.

### 2.8 Immunoblot analyses

Following washes using cold PBS (ATCC 30‐2200), cells were lysed in lysis buffer composed of 150 mmol/L NaCl (pH 7.4) and 1% SDS (Sigma‐Aldrich L4509). 20‐35 µg of protein lysates were resolved via 4%‐15% mini‐PROTEIN TGX gels (Bio‐Rad 456‐1084). Proteins were transferred from the gel to PVDF membranes (Bio‐Rad 170‐4156) using Trans‐Blot Turbo Transfer System (Bio‐Rad). Membranes were blocked using 5% nonfat dry milk (Bio‐Rad 170‐6404) in TBST composed of 1X TBS (Bio‐Rad 170‐6435) with 0.1% Tween (Bio‐Rad 1610781) at ambient temperature for 1 hour. Blocked membranes were incubated with primary antibody overnight at 4°C followed by secondary antibody for 3 hours at ambient temperature. SuperSignal West Chemiluminescent Substrate (Thermo Scientific 34580, 34075, 34095) was applied to the membrane for 5 minutes. ChemiDoc XRS + system (Bio‐Rad) was used to acquire images. Anti‐Ki67 (1:5000 in 5% milk in TBST) and anti‐GAPDH (1:2000 in 5% milk in TBST) were purchased from Abcam (ab92742, ab9485). Anti‐c‐MYC (1:1000 in 5% milk in TBST) was purchased from Santa Cruz (sc‐40). Anti‐HN1 (1:1000 in 5% milk in TBST) was purchased from GeneTex (GTX106585). Anti‐pAMPKα Thr172 (1:1000 in 5% BSA in TBST), anti‐AMPKα (1:1000 in 5% BSA in TBST), anti‐pAKT1 Ser473 (1:1000 in 5% BSA in TBST), anti‐AKT (pan) (1:1000 in 5% milk in TBST), goat anti‐rabbit IgG HRP‐linked (1:1000 in 5% milk in TBST), and goat anti‐mouse IgG HRP‐linked (1:1000 in 5% milk in TBST) were purchased from Cell Signaling Technologies (2535, 2532, 4060, 2967, 7074, 7076). Uncropped versions of all immunoblot images are included in Figure S4A‐Z.

### 2.9 IP‐MS analyses of the JPT1

Twenty million RL95‐2 cells were used per immunoprecipitation. Two independent experiments were performed with two technical replicates per immunoprecipitation. For each immunoprecipitation, 30 µL of protein G sepharose 4 fast flow (GE Healthcare 17061801) was bound to 5 µL of anti‐HN1 (GeneTex GTX106585) for 2.5 hours at 4°C with end‐over‐end rotation. Cells were lysed in 50 mmol/L Tris (pH 7.4), 137 mmol/L NaCL, 10% glycerol, and 0.1% Triton X supplemented with 1 X Halt protease and phosphatase inhibitor with 5 mmol/L EDTA (ThermoFisher Scientific 1861280) for 30 min on ice. Lysate was spun at 16 000 × g for 20 minutes at 4°C and the supernatant was incubated with protein G sepharose 4 fast flow and anti‐HN1 for 3 hours at 4°C with end‐over‐end rotation. Sample was resolved on a 4%‐15% mini‐PROTEAN TGX gel (Bio‐Rad 4561084) and 10 gel bands per lane were cut. Gel bands were digested using trypsin/LysC Mix (Promega V5073) overnight and samples were cleaned up using Reversed‐Phase ZipTip (Millipore ZTC185096) according to the manufacturer's protocol prior to LC‐MS/MS analysis. Immunoprecipitation with anti‐JPT1 and protein G sepharose 4 fast flow was performed in duplicate. Immunoprecipitation with protein G sepharose 4 fast flow alone was used as a control.

### 2.10 Statistics

Significantly altered proteins were identified using edgeR^19^ based on PSMs normalized to the patient sample exhibiting the lowest total PSM counts (edgeR *P* < .05). Significance testing of JPT1 IHC intensity data was performed using Mann‐Whitney U test in MedCalc (version 19.0.7). Spectral count and IHC expression are shown as box and whisker plots. The ends of the box show the upper and lower quartile and the whiskers outside the box extend to the lowest and highest values. The horizontal line inside the box represents the median. GraphPad Prism (version 8.2.0) was used to perform two‐way ANOVA for the cell proliferation data and unpaired *t* test for the gene expression data. Data were log transformed prior to statistical analyses. Bar and line figures for cell proliferation analyses reflect mean and standard deviation of three technical replicate measurements. Integration of JPT1 and MKI67 IHC abundance data was performed by logistic regression analyses in MedCalc. Receiver operator characteristic (ROC) analyses were performed using the method described by DeLong et al^30^ using default settings in MedCalc. For comparison of JPT1 transcript abundance with overall survival in 540 EC patients, normalized RNA‐seq data (TCGA V2)^20^ for *JPT1* and *MKI67* were downloaded from the https://gdac.broadinstitute.org/ and transcript abundance was directly compared by Spearman Rho correlation in MedCalc. Clinical characteristics were extracted from cgdsr (version 1.2.5) and a Kaplan‐Meier analysis with log‐rank testing (*P*‐value <.05 was considered significant) was performed to assess the relationship between *JPT1* abundance and patient outcome using survival (version 2.37‐7) package in R (version 3.1.2). For Kaplan‐Meier curves, high vs low transcript expression was defined by the median cut‐point capped at 60 months.

## 3 RESULTS

### 3.1 Proteomic analysis of endometrial cancer (EC) tumor tissues collected from pre‐ and post‐treated patient reveals conserved protein alterations between metformin responders and nonresponders.

**Figure 1:**
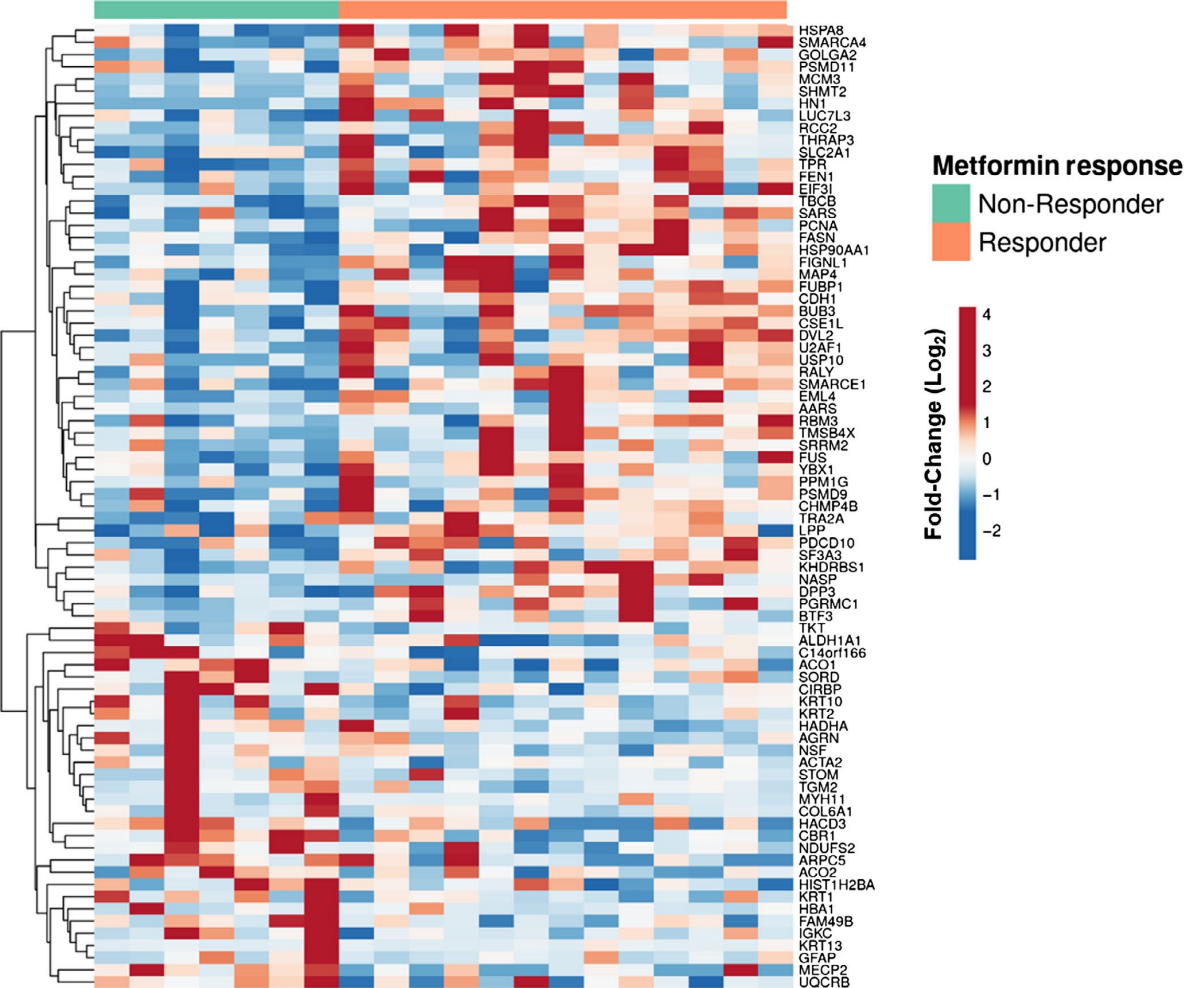
Differential proteomic analyses of endometrial cancer (EC) tissues collected from women who responded (n = 13) or did not respond (n = 7) to metformin treatment. Heatmap details a supervised analyses of 79 proteins (edgeR *P* < .05) significantly altered between EC patients who did or did not respond to metformin treatment. Heatmap was generated using ClustVis (https://biit.cs.ut.ee/clustvis/)

Tumor tissues from EC patients in a preoperative window trial were stratified as responders (n = 13) or nonresponders (n = 7) to metformin treatment. Response was defined as a decrease in IHC staining for MKI67 when comparing pre‐ vs posttreatment EC tissue as previously described.^4^ Quantitative LC‐MS/MS‐based global proteomic analyses of pathologically defined tumor cell populations harvested by LMD from FFPE endometrial biopsies and EC surgical tumor tissues identified 1289 proteins by at least two PSMs across patients (Table S1 and S2). Seventy‐nine proteins were identified to be significantly altered (edgeR *P*‐value ≤.05) in pretreatment tumor cells from metformin responder and nonresponder patients (Figure 1; Table [Link], [Link]). Pathway analysis revealed top altered pathways to include PRKAA2, also known as AMPK signaling (Table 1; Table S4A), along with those related to activating cellular signaling, regulating cellular proliferation, and inhibiting cell death and apoptosis in tissues in metformin responders (Table 1, Table S4B). Protein alterations in pretreatment tissues from responder vs nonresponder patients (Table S5A) were correlated with significant alterations in post‐ vs pretreated tissues from metformin responders (edgeR *P*‐value ≤.05), revealing 11 co‐altered proteins between these groups (Uniprot Accession * and bolded, Table S5A). Further analyses of metformin responders revealed activation of cell death and apoptosis signaling, but inhibition of viral infection as well as molecular transport in response to metformin (Table S5B). Additional analyses of significant protein alterations in metformin nonresponders (Table S6A) also revealed activation of cell death as well as organ hypoplasia signaling, inhibition of T cell proliferation, and leukocyte viability signaling in post‐ vs pre‐metformin‐treated tissues (Table S6B).

### 3.2 Jupiter microtubule‐associated homolog 1—a predictive and pharmacodynamic biomarker of metformin response in endometrial cancers

**Figure 2:**
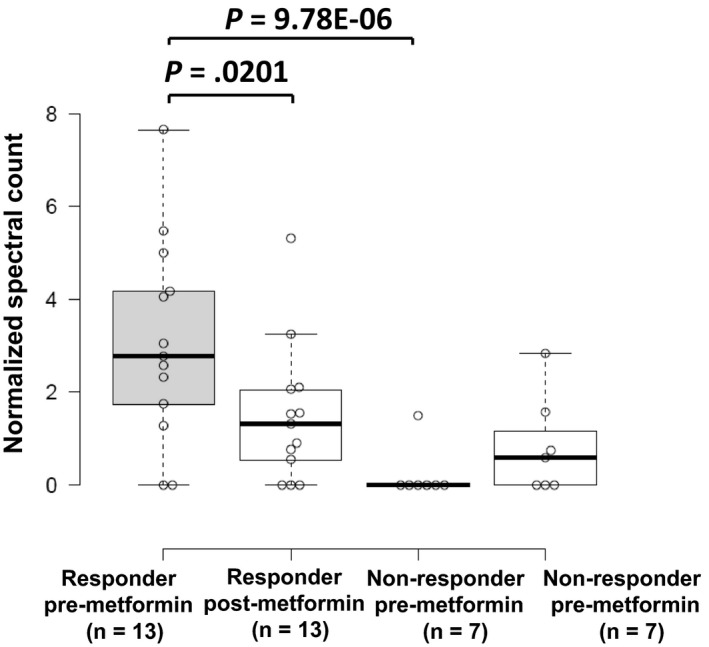
Jupiter microtubule‐associated homolog 1 (JPT1) protein is elevated in metformin responders and decreases following metformin treatment. JPT1 was observed as significantly abundant in metformin responder vs nonresponder patients and was significantly decreased in abundance following metformin treatment in responders by LC‐MS/MS‐based proteomic analyses (*P*‐values calculated using edgeR testing). The horizontal line inside the box represents the median. The limits of the box show the upper and lower quartiles and the whiskers correspond to the minimum and maximum values observed

**Figure 3:**
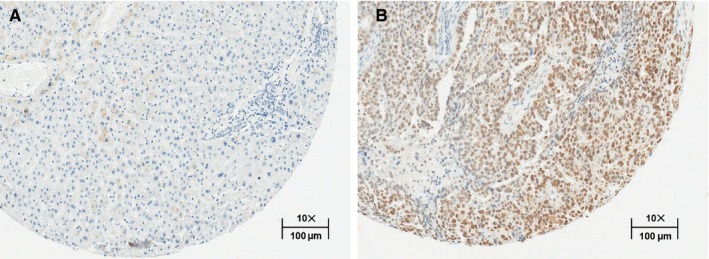
JPT1 immunohistochemical analyses. IHC staining in EEC tissues harvested from a representative (B) responder vs (A) nonresponder patient, pre‐metformin treatment. Images reflect subregions of TMA tissue spots imaged at 10× at a scale of 100 µm; collected reproduced from Leica Web Viewer v12.4

**Figure 4:**
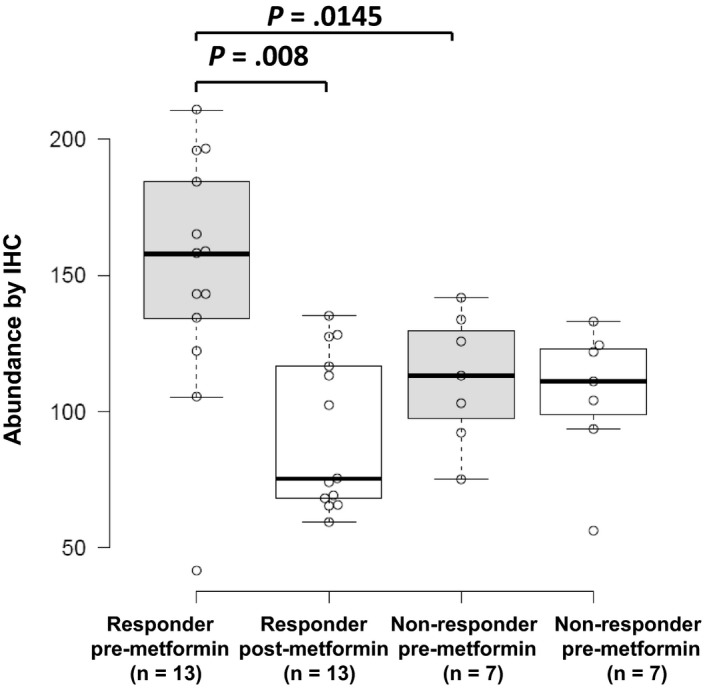
Verification of altered JPT1 protein abundance by immunohistochemical analyses of EEC patients' tissues collected from metformin responder (n = 13) vs nonresponders (n = 7). IHC H‐Score for JPT1 abundance in metformin responders and nonresponder, pre‐ and post‐metformin treatment (*P*‐values calculated using Mann‐Whitney *U* test). The horizontal line inside the box represents the median. The limits of the box show the upper and lower quartiles and the whiskers correspond to the minimum and maximum values observed

Among proteins correlating with response, we prioritized JPT1 for further validation as this protein exhibited the greatest fold‐change difference in pre‐metformin treatment biopsies from responders vs nonresponders (log_2_ ratio = 3.84, edgeR *P*‐value = 9.78E−06), and was decreased in abundance in post‐ vs pretreated tumor tissues in metformin responders (log_2_ ratio = −1.06, edgeR *P*‐value = .0201), but remaining largely unaltered in nonresponders (Figure 2). Immunohistochemical staining for JPT1 in the same set of patient tissues verified the proteomics data (Figures 3, 4; Table S7). These results confirmed that JPT1 expression is significantly elevated in responder vs nonresponder tissues, pre‐metformin treatment (*P*‐value = .0145) and that JPT1 expression was decreased in responders, but unaltered in nonresponders, post‐metformin treatment (*P*‐value = .008) (Figure 4).

### 3.3 Jupiter microtubule‐associated homolog 1 expression is decreased following metformin treatment of endometrial cancer cells, but HN1 is not necessary for response to metformin or for endometrial cancer cell proliferation

**Figure 5:**
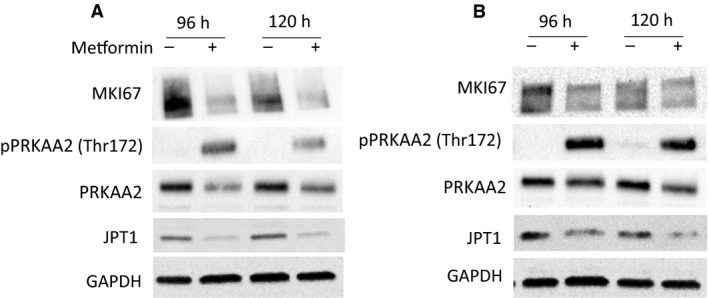
JPT1 decreases in response to metformin treatment in endometrial cancer cells. A, RL95‐2 and (B) ACI‐181 cells were treated with metformin (20 mmol/L) for 96 h or 120 h and equivalent amounts of protein lysate were immunoblotted for JPT1 protein abundance as well as PRKAA2, p‐ PRKAA2 (Thr172), and MKI67 proteins. Uncropped immunoblots are shown in Figure S4E‐J

**Figure 6:**
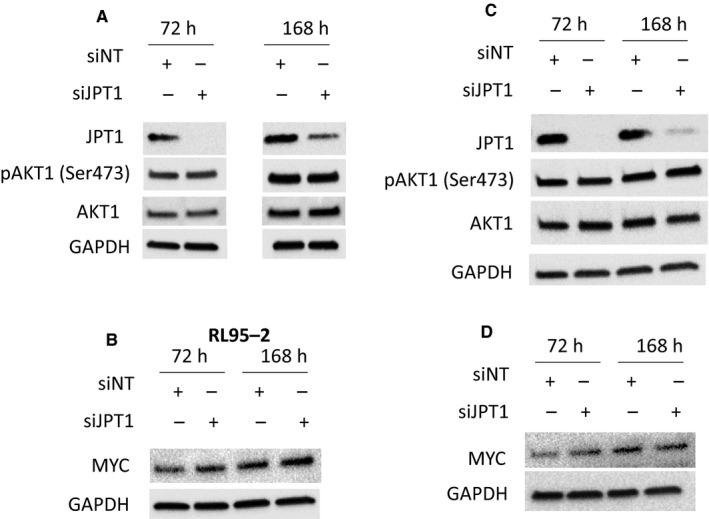
Immunoblots of RL95‐2 and ACI‐181 demonstrating siJPT1 knockdown. JPT1 expression was knocked down in RL95‐2 (A and B) and ACI‐181 (C and D) cells by small interfering RNA (siRNA) targeting JPT1 or with a nontargeting (siNT) control and confirmed at the protein (A and B) level before (72 h) and following (168 h) dose‐response analyses with metformin treatment. Please note that assessment of MYC abundance in RL‐95‐2 was generated on a different immunoblot than JPT1, pAKT (S473), and total AKT, but identical protein lysates were used for all RL‐95‐2 immunoblots. Uncropped immunoblots are shown in Figure S4K‐T and aa

**Figure 7:**
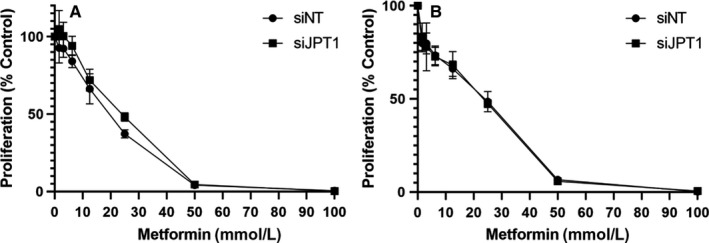
JPT1 knockdown does not alter the response of endometrial cancer cell lines to metformin. RL95‐2 (A) and ACI‐181 (B) cells were treated with metformin 72 h following siRNA transfection and dose‐response was assessed after an additional 72 h by MTS assay. Proliferation (% control) data represent three technical replicates from one biological sample. Error bars represent standard deviation. Two‐way ANOVA was performed after log transformation of the data (*P*‐value was .386639 and .979984 for RL95‐2 and ACI‐181, respectively)

**Figure 8:**
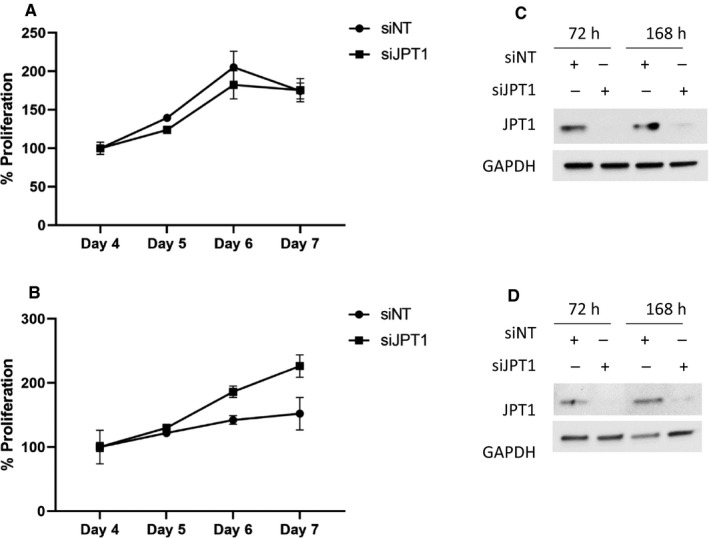
JPT1 knockdown does not alter the proliferation of endometrial cancer cell lines. JPT1 expression was silenced in ACI‐181 (A, C) and RL95‐2 (B, D) cells by small interfering RNA (siRNA) targeting JPT1 or with a nontargeting (siNT) control and confirmed at the protein level (B & D) before (72 h) and following (168 h) daily assessment of cellular proliferation by MTS assay (5000 cells were seeded per well). Each data point reflects triplicate technical replicates measured per day with error bars reflecting standard deviation. Data were log transformed and analyzed by two‐way ANOVA (*P*‐value was .09319 and .333719 for RL95‐2 and ACI‐181, respectively). Uncropped immunoblots are shown in Figure S4U‐X

The EC cell lines, RL95‐2 and ACI‐181, were treated with 20 mmol/L metformin (~LD50) for 96 and 120 hours, and JPT1, AMPKα, p‐AMPKα (T172), and MKI67 protein abundance were assessed by immunoblotting. Metformin induced activation of AMPKα, as evidenced by an increase in p‐AMPKα (T172) abundance and further mediated a decrease in MKI67 and JPT1 abundance (Figure 5). We then assessed the impact of JPT1 on response to metformin in EC cells in which JPT1 expression was silenced by siRNAs targeting *JPT1* mRNA (Figure 6, Figure S1). These analyses revealed that loss of JPT1 expression did not alter the response of EC cells to metformin treatment (Figure 7, Figure S2A,B). As AKT1 has previously been noted to be hyperactivated in low JPT1 backgrounds,^21^ we further assessed the activation of AKT1 in JPT1‐silenced cells. However, we did not observe alterations of p‐AKT1 (S473) abundance in EC cells transfected with JPT1‐specific vs nontargeting siRNAs (Figure 6). Further, recent evidence has shown that JPT1 knockdown results in decreased abundance of the *MYC* oncogene.^22^ We further assessed MYC protein abundance in JPT1‐silenced EC cells, but did not reproduce this finding (Figure 6). We further assessed the impact of silencing JPT1 on the proliferation rate of RL95‐2 and ACI‐181 cells (Figure 8, Figure S3). These analyses revealed that loss of JPT1 expression does not significantly alter the proliferation of EC cells.

### 3.4 Jupiter microtubule‐associated homolog 1 is correlated with MKI67 expression and associated with altered overall survival in endometrial cancer patients

As MKI67 and JPT1 abundance were both decreased following metformin treatment, we assessed correlation trends of JPT1 protein abundance (Table S7) relative to MKI67 protein abundance previously assessed in these tissues.^4^ We found that JPT1 and MKI67 exhibit concordant abundance trends in comparisons of both pre‐ (Spearman = 0.44, *P* = .0515) as well as in post‐ (Spearman = 0.58, *P* = .0075) metformin‐treated tissues. We further compared the performance of JPT1 to predict response to metformin vs MKI67 by ROC analyses (Figure S5A). We found that JPT1 (AUC > 0.825) exhibits comparable performance to MKI67 (AUC > 0.825) as a biomarker of response to metformin (JPT1 vs MKI67 ROC *P* > .05, ie, nonsignificant difference). We also found that JPT1 exhibited comparable performance of JPT1 and MKI67 combined (JPT1 vs JPT1&MKI67 ROC *P* > .05, ie, nonsignificant difference). We observe similar performance for JPT1 (AUC = 0.87) and MKI67 (AUC = 0.873) as pharmacodynamic markers of metformin response (JPT1 vs JPT1&MKI67 ROC *P* > .05, ie, nonsignificant difference, Figure S5B). We further assessed the correlation of *JPT1* and *MKI67* transcript expressions using a public RNA‐seq dataset established from 540 EC patient's tumor tissues,^20^ and found that these genes were positively correlated in EC patient tissues (Spearman = 0.37, *P* < .0001, Figure S6A). We also assessed whether *JPT1* abundance was directly correlated with disease outcome in EC patients and found that elevated expression of *JPT1* was associated with poor overall survival (n = 540 EC patients, HN1 high vs low tertile abundance, HR = 2.045, 95% CI = 1.148‐3.64, Univariate Cox *P* = .015, Log‐rank *P*‐value = .047, Figure S6B), but not with disease‐free survival (n = 499 EC patients, Log‐Rank *P*‐value = .14, data not shown).

### 3.5 Jupiter microtubule‐associated homolog 1 exhibits binding partners regulating metabolic signaling pathways in endometrial cancer cells

We performed an immunoprecipitation and mass spectrometry (IP‐MS)‐based analysis of JPT1 in sub‐confluent RL95‐2 cells to identify putative binding partners of JPT1 (Table S8A). JPT1 was the most abundantly identified protein in JPT1 IP‐MS biological replicate analyses, that is, 61 ± 8 PSMs, following removal of proteins identified in a companion control IP‐MS analyses of sepharose beads alone that contained no anti‐JPT1 antibody. Top functional pathways enriched following the analyses of proteins abundantly identified with JPT1, that is, ≥10 PSMs in both JPT1 IP‐MS analyses (designated with * in Table S8A), were associated with metabolic pathways such as purine nucleotides de novo biosynthesis, glycolysis, and gluconeogenesis as well as fatty acid biosynthesis signaling (Table S8B).

## 4 DISCUSSION

The current study sought to identify proteomic alterations in tumor tissues harvested from EC patients before and after daily treatment with metformin (850 mg) for 4 weeks prior to surgical staging. Women who responded to metformin exhibited a significant decrease (7 to 50%) in the expression of the cellular proliferation marker MKI67 in post‐treated tumor tissues, whereas nonresponders exhibited a slight increase in MKI67 IHC staining (~2%‐12%), as described previously.^4^ A global quantitative LC‐MS/MS‐based proteomics analysis identified discrete protein alterations in tumor cells harvested by LMD from pretreated endometrial biopsies that correlate with canonical cellular pathways that include PRKAA2 signaling, a kinase regulating cellular energy homeostasis activated by metformin,^6^ as well as pathways involved in activating cellular proliferation and inhibiting cell death signaling in metformin responders. Among the proteins altered, JPT1 was the most significantly elevated protein in pretreated endometrial biopsies from metformin responders (responder vs nonresponder logFC = 3.42, edgeR *P*‐value = 9.78E−06). We cross‐correlated JPT1 in pre‐ and posttreatment biopsies in metformin responders and found that JPT1 was also significantly decreased following metformin treatment (post‐ vs pretreatment logFC = −1.06, edgeR *P*‐value = 0.02)—a finding that was independently verified by IHC.

JPT1, also known as HN1, is a ~16 kDa protein (Q9UK76, uniprot.org) initially identified as an abundant transcript in both murine hematopoietic and brain cells.^23^ As JPT1 was significantly elevated in patients who responded to metformin, we assessed the impact of silencing JPT1 expression on response to metformin using two EC cell line models, that is, RL95‐2 and ACI‐181 (Figure 6). We found that loss of JPT1 did not alter cellular response to metformin treatment in vitro. In cancer, JPT1 has been shown to inhibit the growth of androgen‐sensitive, but promote the growth of androgen‐insensitive, prostate cancer cells through altered androgen receptor stability and by modulating cell metabolism and cell cycle progression through AKT serine/threonine kinase 1 (AKT1) and downstream glycogen synthase kinase 3 beta (GSK3B)/ catenin β1 (CTNNB1)‐dependent mechanisms.^21^, ^24^ Specifically, silencing of JPT1 expression increased the activation of AKT1 via upregulation of p‐AKT1 (S473) in androgen‐dependent and ‐independent prostate cancer cell lines.^21^ We similarly assessed modulation of p‐AKT1 (S473) following knockdown of JPT1 in EC cell lines, though we did not reproduce this finding (Figure 5). Recent evidence has also shown that elevated JPT1 expression is directly correlated with poor prognosis in breast cancer patients.^22^ Further investigations revealed that JPT1 increases the migratory and invasive potential of breast cancer cells by upregulating the *MYC* oncogene and downstream effectors in vitro and further increases breast tumorigenesis in vivo.^22^ We, however, did not see any alterations in MYC protein abundance following silencing of JPT1 in EC cells (Figure 5). We found that metformin treatment inhibited EC cell proliferation in vitro, as evidence by decreased MKI67 levels, and that this response was further accompanied by the loss of JPT1 abundance (Figures 6, 7). Pathway analyses of significantly altered proteins in metformin pretreatment tumors from responders revealed that cellular proliferation signaling was altered in metformin responders as evidenced by elevation of proliferating cell nuclear antigen in responder patients (PCNA, edgeR LogFC = 1.29, *P*‐value = .025). PCNA regulates DNA replication via interactions with DNA polymerase delta, and is a marker of cellular proliferation often assessed during routine histopathology analyses of tumor tissues along with MKI67 to determine the proliferative indices of tumor cells in situ.^25^ Indeed, we previously observed^4^ that pretreatment tumor tissues from metformin responders exhibited elevated MKI67 expression levels vs nonresponders (47.3% vs 24.9%, *P*‐value = .004), providing suggestive evidence that these patients may harbor tumor cells with a greater proliferative capacity. That we do not observe an association between JPT1 and response to metformin and as JPT1 levels closely parallel PCNA levels in responders vs nonresponders, JPT1 abundance may also serve as a surrogate measure of proliferative activity in EC cells. Our ROC analyses of JPT1 protein abundance to predict response to metformin revealed similar performance as MKI67, providing evidence to support this hypothesis. Similar to our findings with JPT1, recent assessments of MKI67 have identified that this hallmark biomarker of cellular proliferation does not directly contribute to tumor cell proliferation,^26^, ^27^ but rather functions to maintain cancer stem cell populations^27^ and facilitates chromosomal motility and mitotic spindle interactions.^28^ To gain further insight into the possible biologic roles of JPT1 in EC cells, we performed an immunoprecipitation‐mass spectrometry‐based analyses of JPT1 in sub‐confluent RL95‐2 cells to identify functional binding partner candidates. These analyses revealed putative JPT1‐binding proteins to be predominantly associated with metabolic signaling pathways such as nucleotide and aerobic glycolysis and fatty acid signaling. Future studies should focus on validating these JPT1‐binding partner candidates and elucidating the roles of these targets along with JPT1 in the regulation of metabolic signaling pathways as they pertain to metformin treatment.

Immunohistochemical markers of metformin response used in clinical trials to date include assessments of PRKAA2 abundance and activation state (p‐PRKAA2, T172), mTOR signaling, such as p‐RPS6 kinase as well as p‐EIF4BP1, as well as assessing markers of cellular proliferation.^29^ Indeed, we observe activation of PRKAA2 via increased levels of p‐PRKAA2 (T172) in EC cells treated with metformin in vitro (Figure 5). We did not, however, observe elevated p‐PRKAA2 (T172) by IHC in EC tissues following metformin treatment,^4^ though MKI67 was clearly altered in these same tissues, recapitulating our findings in vitro. Comparative analyses of MKI67 and JPT1 protein abundance by IHC analyses in pre‐ and post‐metformin‐treated patient tissues revealed these protein abundances to be positively correlated. We assessed the correlation of *JPT1* and *MKI67* mRNA abundance in public data for over 500 EC patients and found that *JPT1* and *MKI67* expressions are positively correlated (*R* ≥ .44). Furthermore, we also identified that elevated *JPT1* is associated with poor overall survival, underscoring that high JPT1‐expressing patients may experience a survival benefit from metformin treatment. Limitations of this study include the restricted sample size for our discovery analyses as well as our discovery cohort encompassing obese EC patients. Further validation efforts in an expanded cohort of obese and nonobese, pre‐ and post‐metformin‐treated EC tissue biopsies will be the focus of future efforts to support suggestive evidence that IHC assessment of JPT1 in presurgical endometrial tissue biopsies may aid to prioritize patients for neoadjuvant treatment with metformin, and that JPT1 may further function as a pharmacodynamic surrogate of therapeutic response to metformin.

## 5 DISCLAIMER

The views expressed herein are those of the authors and do not reflect the official policy of the Department of Army/Navy/Air Force, Department of Defense, Henry M. Jackson Foundation for the Advancement of Military Medicine, or US Government. The authors published a preprint version of this article in bioRxiv on 4/9/2019, (i.e.) https://doi.org/10.1101/602870.

## CONFLICT OF INTEREST

The authors report no conflict of interest.
