# Supplementary figures and images for: Jupiter microtubule‐associated homolog 1 (JPT1): A predictive and pharmacodynamic biomarker of metformin response in endometrial cancers
Source: Cancer Med. 2019 Dec 6;9(3):1092–103. doi: 10.1002/cam4.2729 (PMC6997075; doi:10.1002/cam4.2729)

Supplemental Figure 1.

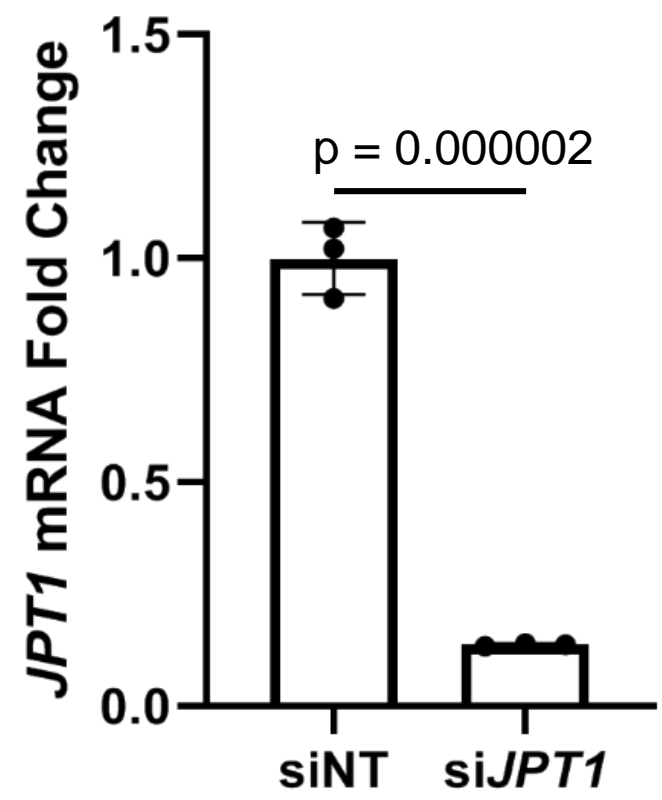

Supplement: Supplementary file 1 [file CAM4-9-1092-s001.pdf]

Supplemental Figure 2a.

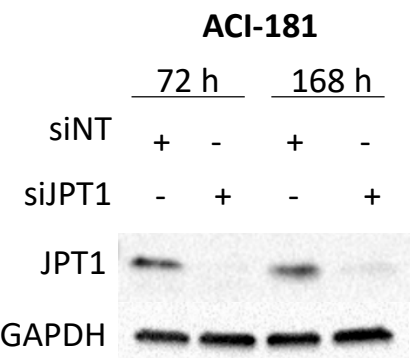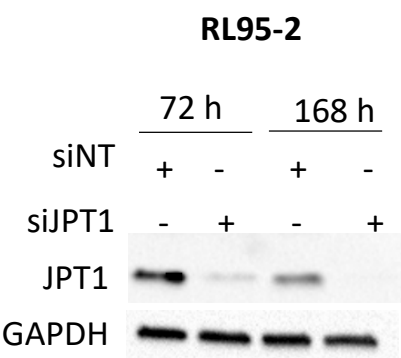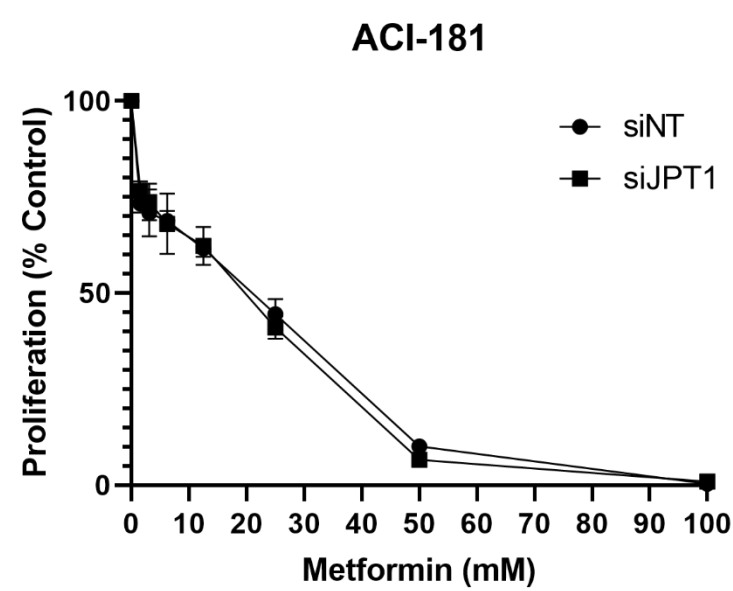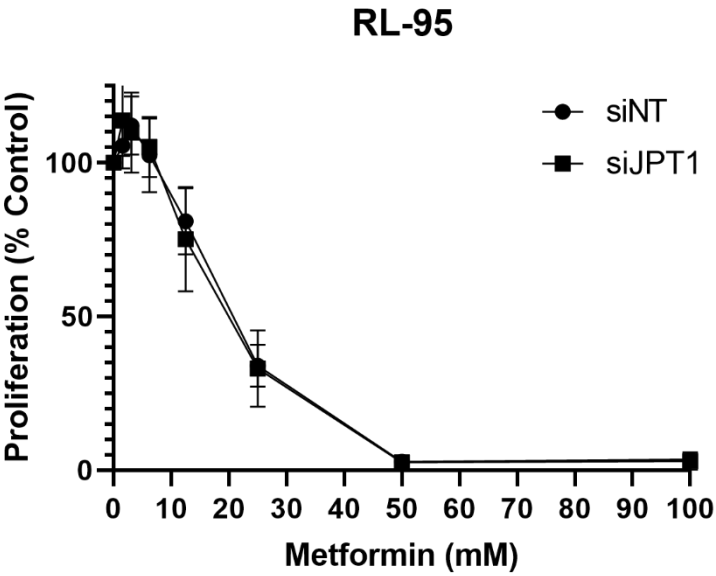

Supplemental Figure 2b.

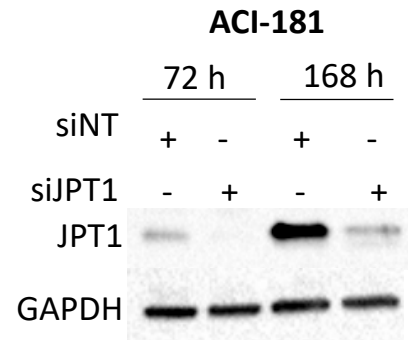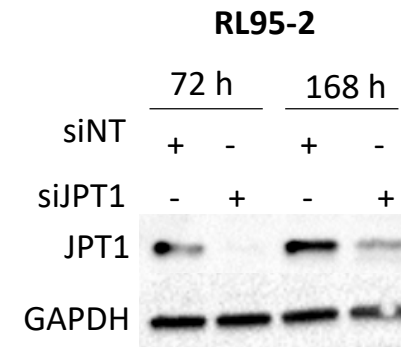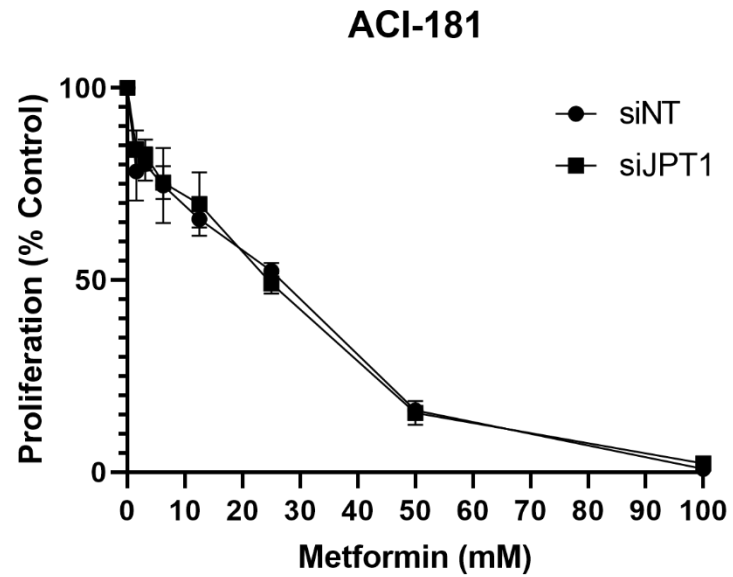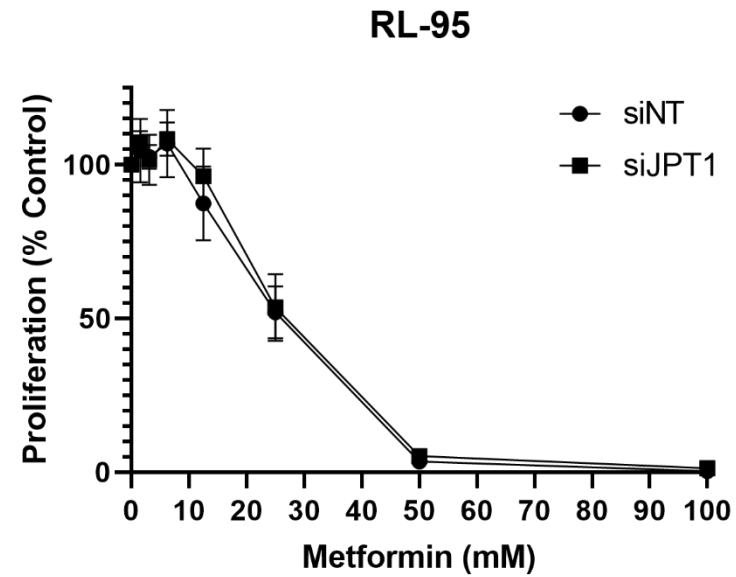

Supplement: Supplementary file 2 [file CAM4-9-1092-s002.pdf]

Supplemental Figure 3.

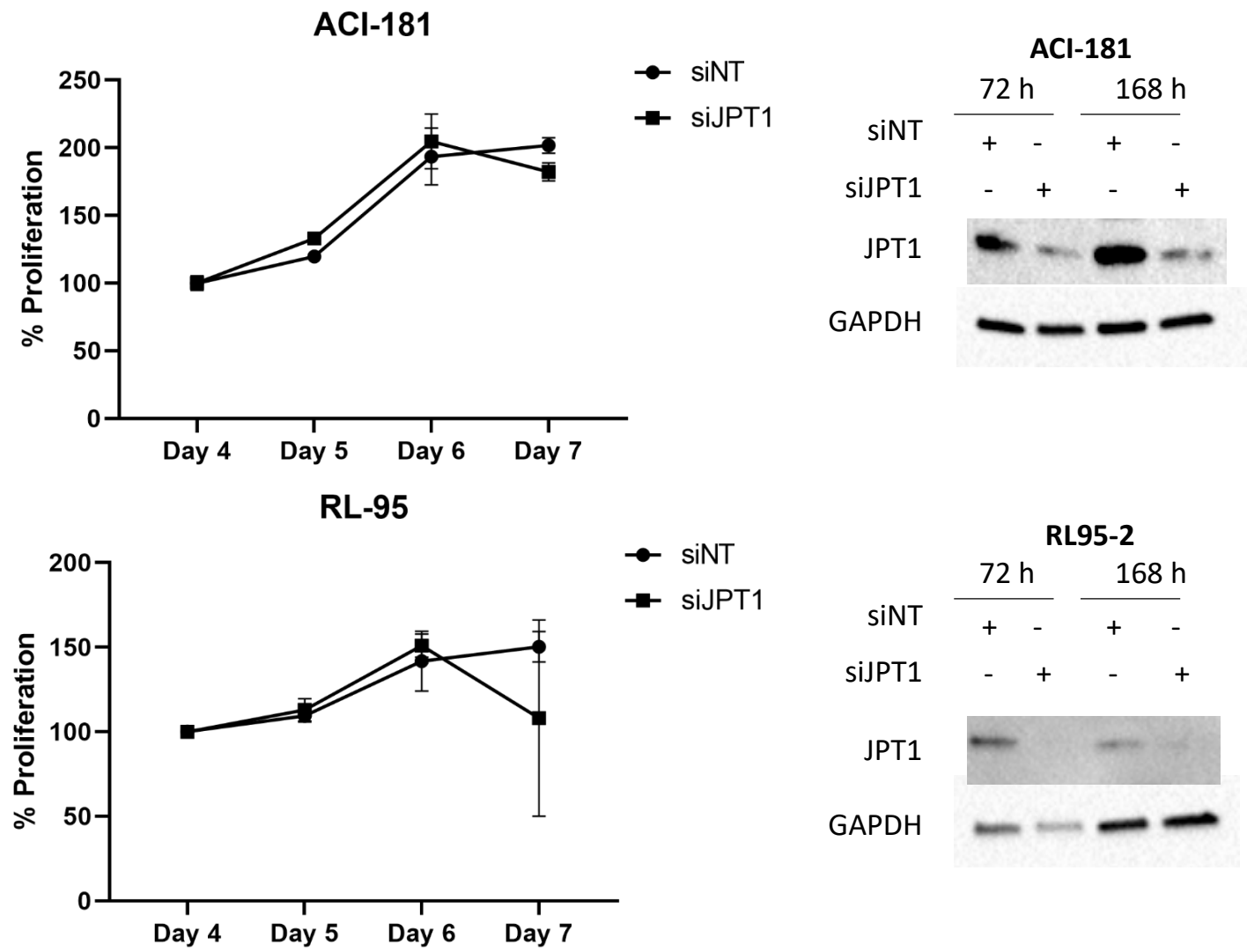

Supplement: Supplementary file 3 [file CAM4-9-1092-s003.pdf]
