# Supplementary material for: Jupiter microtubule‐associated homolog 1 (JPT1): A predictive and pharmacodynamic biomarker of metformin response in endometrial cancers
Source: Cancer Med. 2019 Dec 6;9(3):1092–103. doi: 10.1002/cam4.2729 (PMC6997075; doi:10.1002/cam4.2729)
Supplement: Supplementary file 4 [file CAM4-9-1092-s004.pdf]

Supplemental Figure 4a.

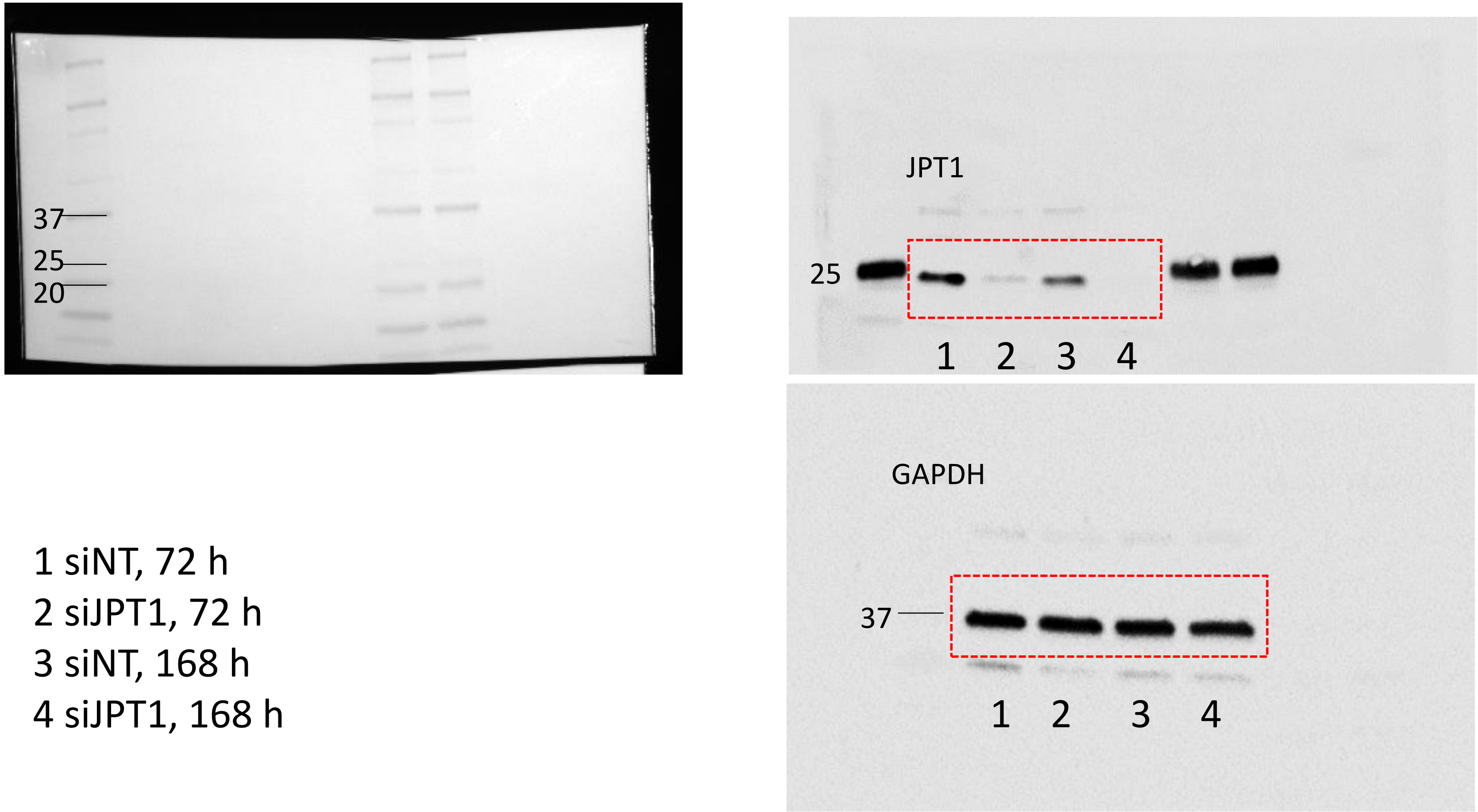

1 siNT, 72 h
2 siJPT1, 72 h
3 siNT, 168 h
4 siJPT1, 168 h

Supplemental Figure 4b.

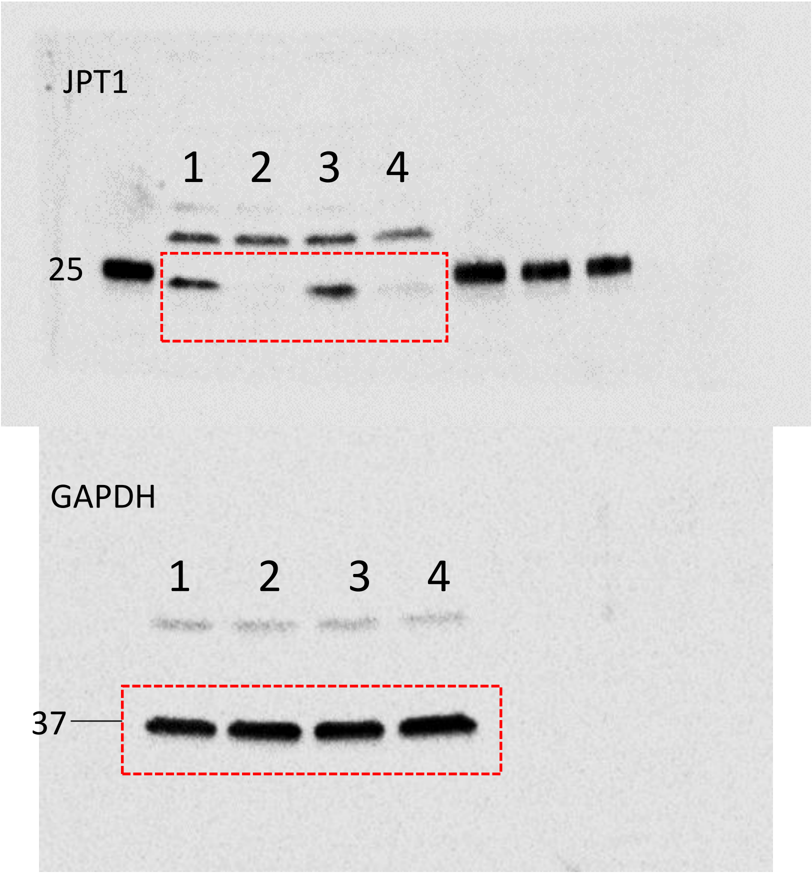

1 siNT, 72 h
2 siJPT1, 72 h
3 siNT, 168 h
4 siJPT1, 168 h

Supplemental Figure 4c.

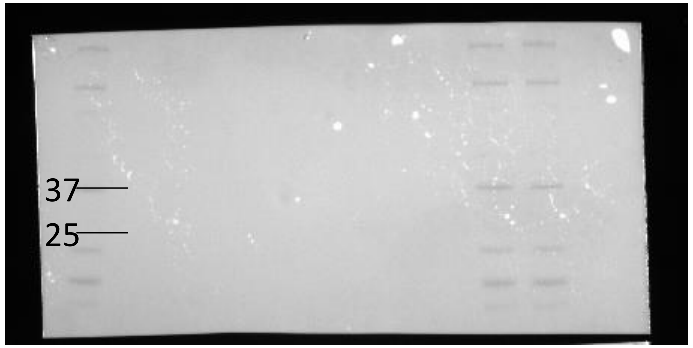

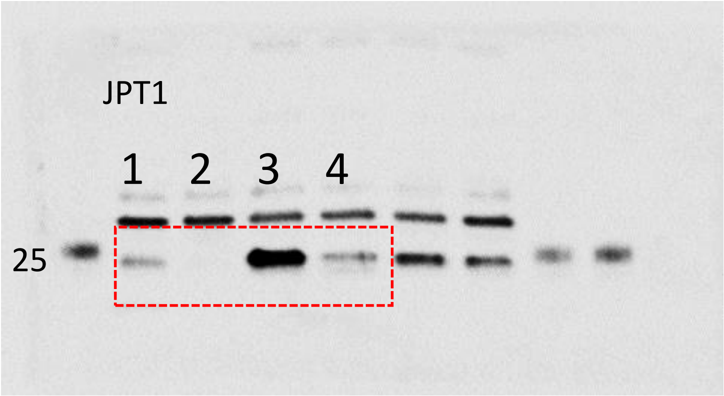

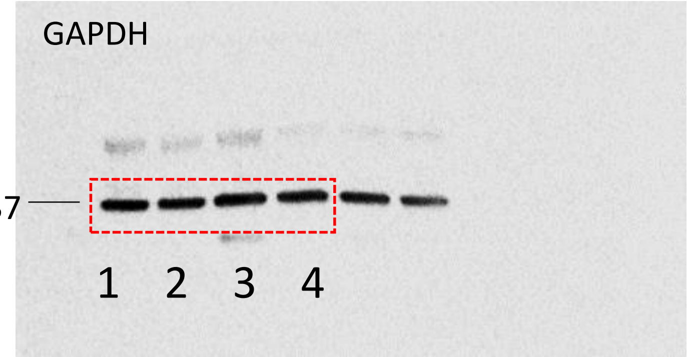

1 siNT, 72 h
2 siJPT1, 72 h
3 siNT, 168 h
4 siJPT1, 168 h

Supplemental Figure 4d.

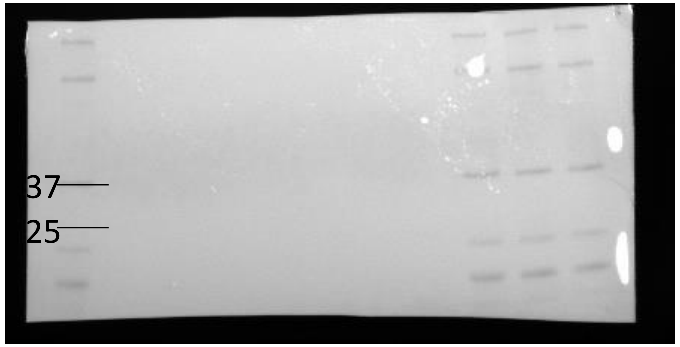

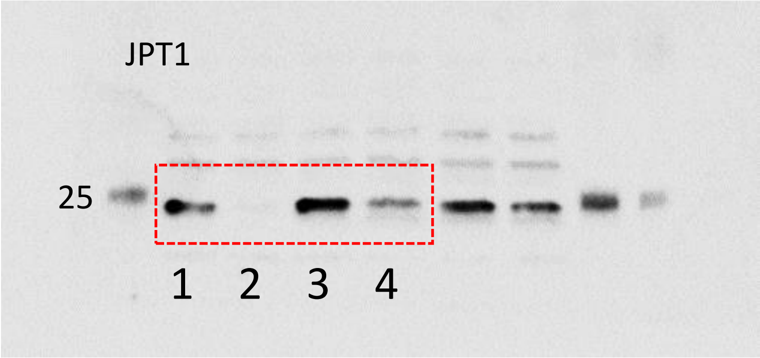

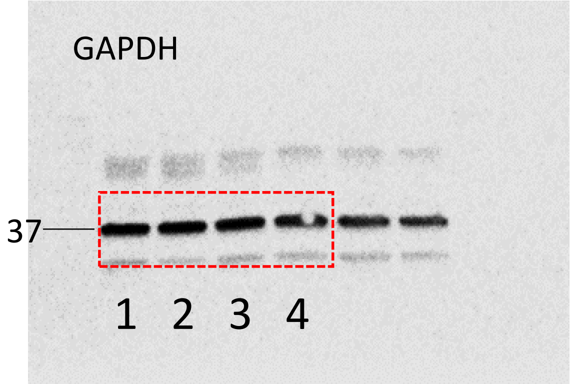

1 siNT, 72 h
2 siJPT1, 72 h
3 siNT, 168 h
4 siJPT1, 168 h

Supplemental Figure 4e.

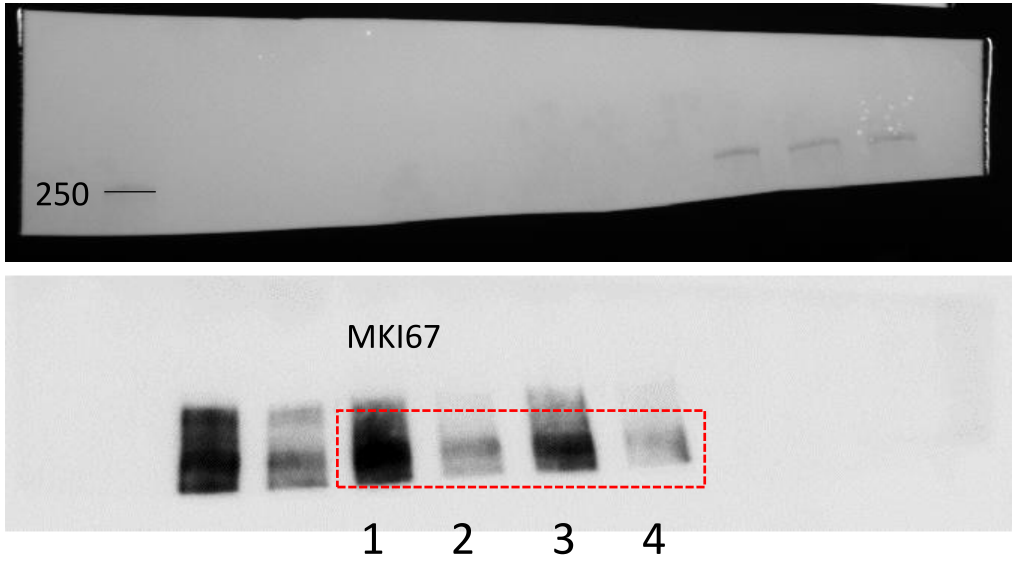

1 - Metformin, 96 h
2 + Metformin, 96 h
3 - Metformin, 120 h
4 + Metformin, 120 h

Supplemental Figure 4f.

p-PRKAA2 (Thr172)

total PRKAA2

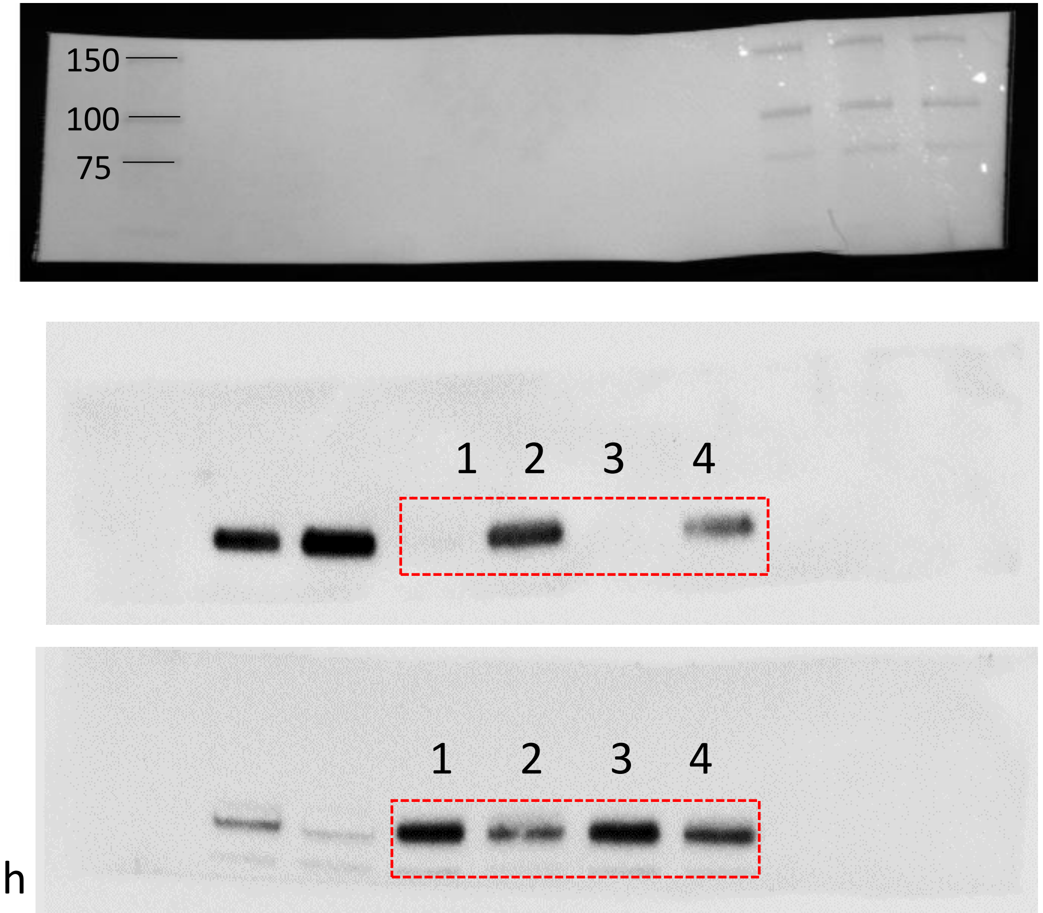

1 - Metformin, 96 h
2 + Metformin, 96 h
3 - Metformin, 120 h
4 + Metformin, 120 h

Supplemental Figure 4g.

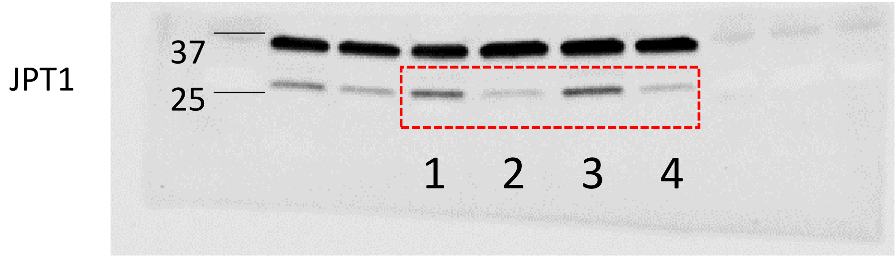

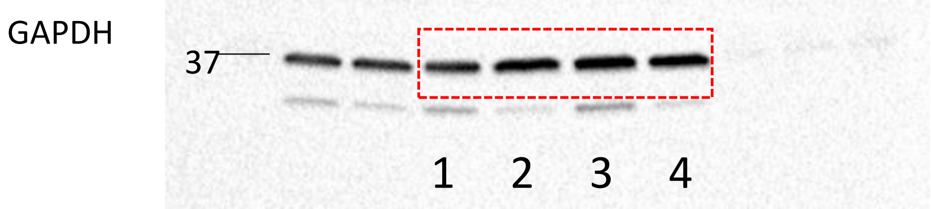

1 - Metformin, 96 h
2 + Metformin, 96 h
3 - Metformin, 120 h
4 + Metformin, 120 h

Supplemental Figure 4h.

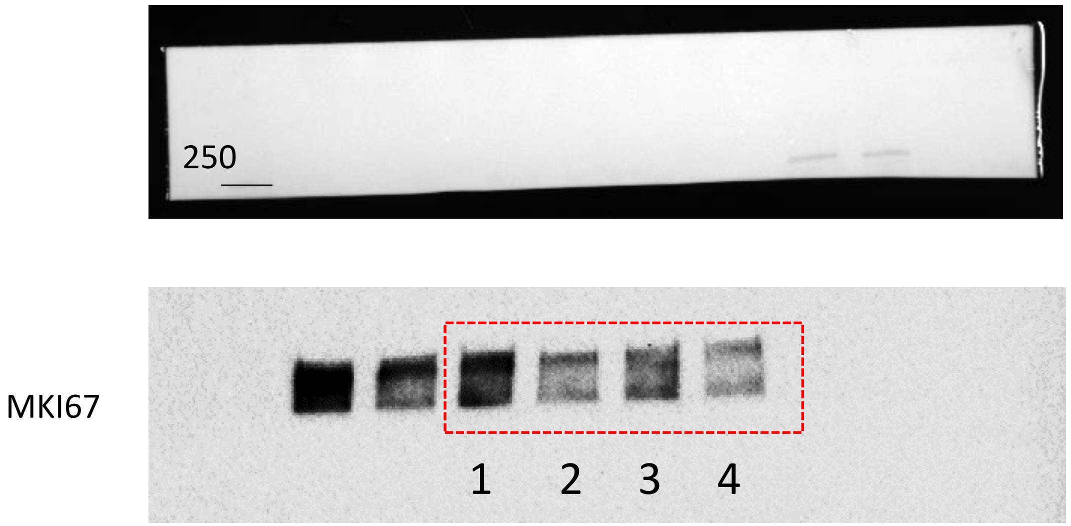

1 - Metformin, 96 h
2 + Metformin, 96 h
3 - Metformin, 120 h
4 + Metformin, 120 h

Supplemental Figure 4i.

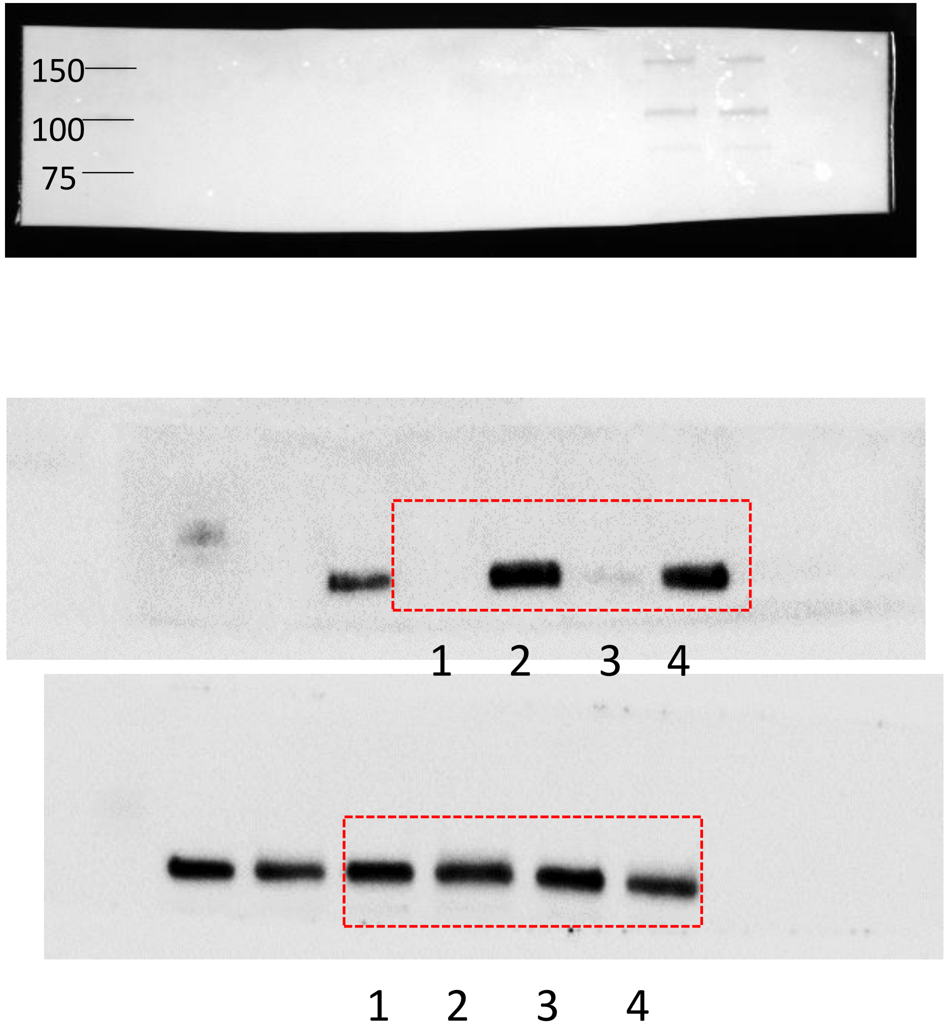

p-PRKAA2 (Thr172)

total PRKAA2

1 - Metformin, 96 h
2 + Metformin, 96 h
3 - Metformin, 120 h
4 + Metformin, 120 h

Supplemental Figure 4j.

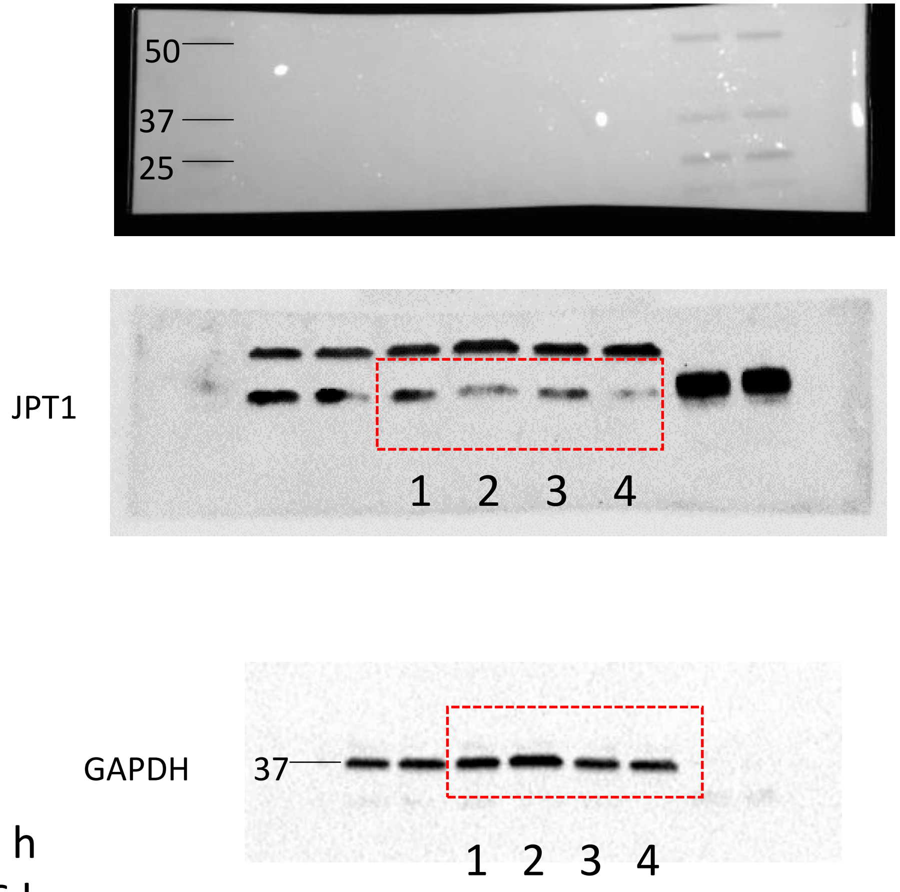

1 - Metformin, 96 h
2 + Metformin, 96 h
3 - Metformin, 120 h
4 + Metformin, 120 h

Supplemental Figure 4k.

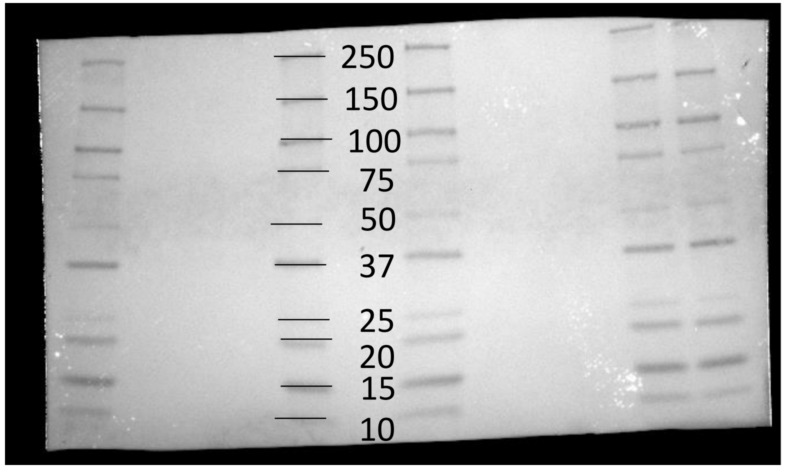

JPT1

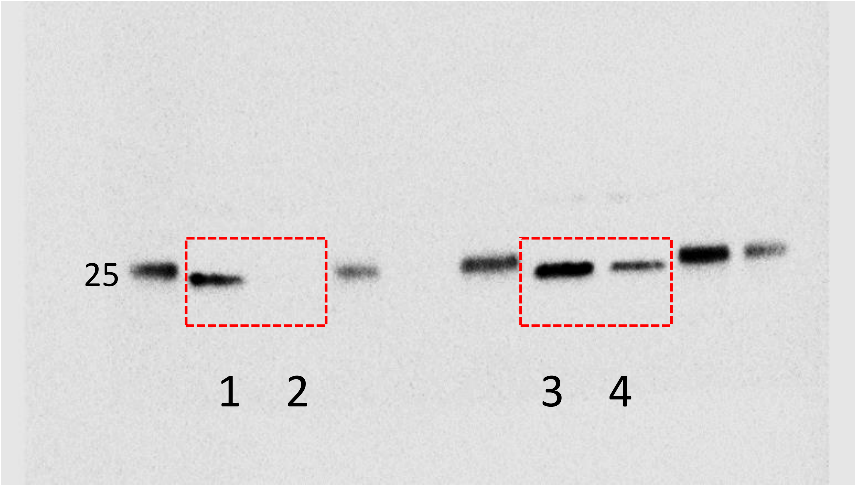

1 siNT, 72 h
2 siJPT1, 72 h
3 siNT, 168 h
4 siJPT1, 168 h

Supplemental Figure 4l.

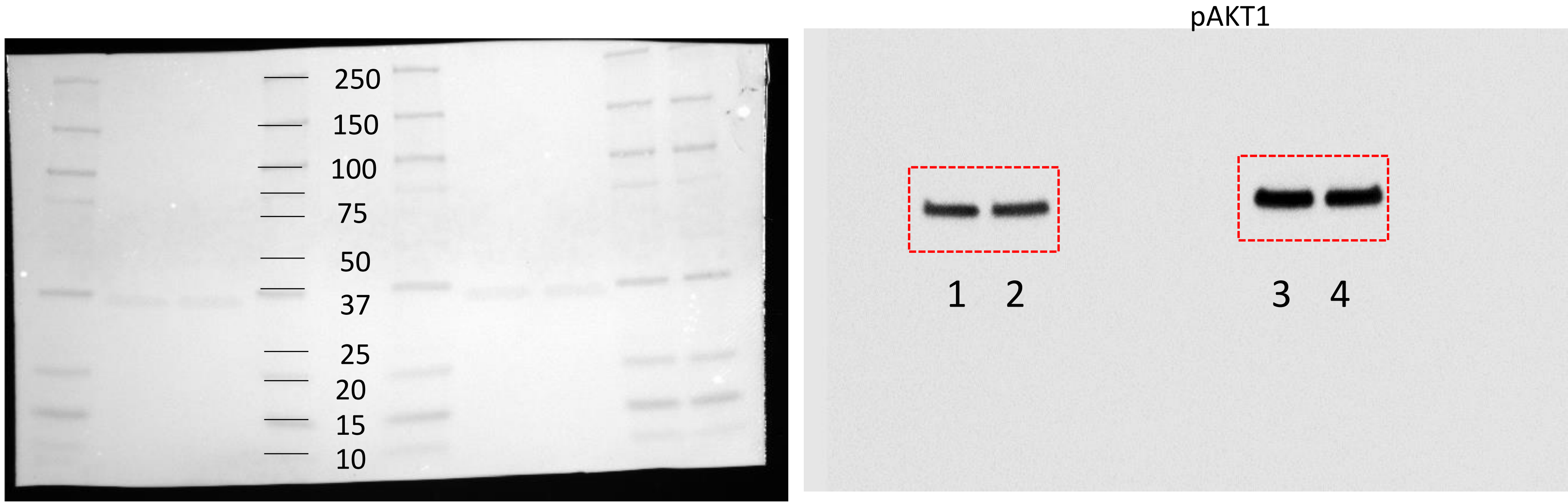

1 siNT, 72 h
2 siJPT1, 72 h
3 siNT, 168 h
4 siJPT1, 168 h

Supplemental Figure 4m.

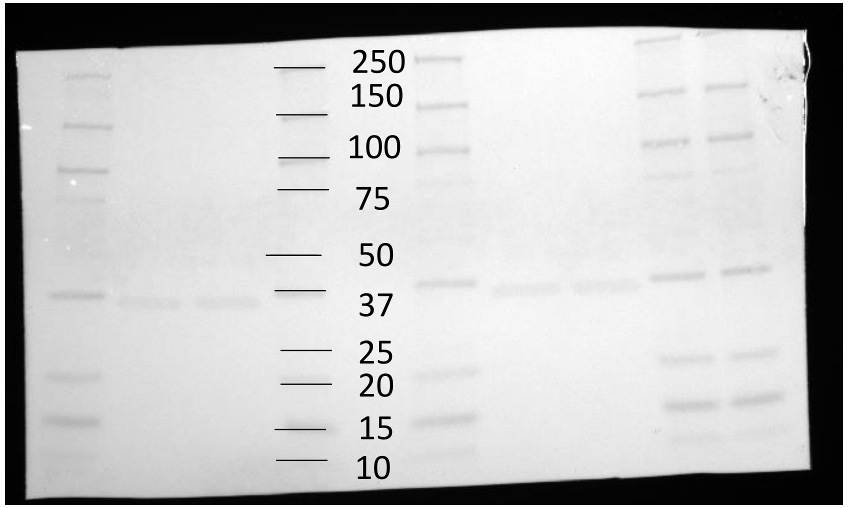

Total AKT1

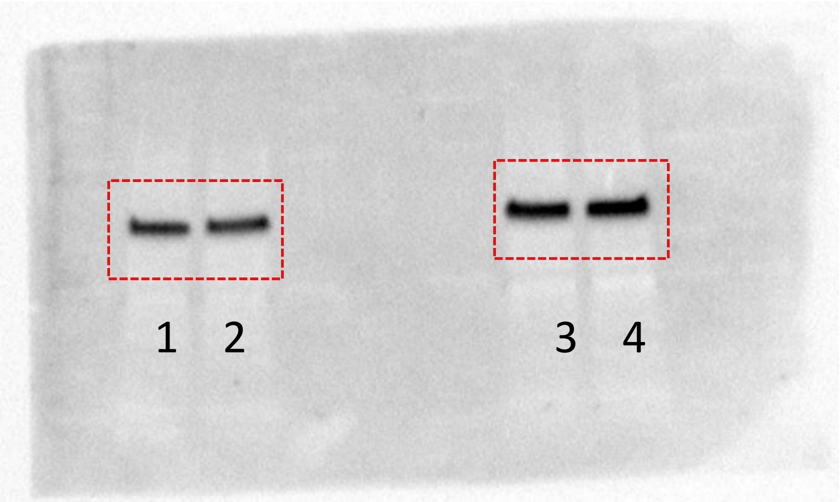

1 siNT, 72 h
2 siJPT1, 72 h
3 siNT, 168 h
4 siJPT1, 168 h

Supplemental Figure 4n.

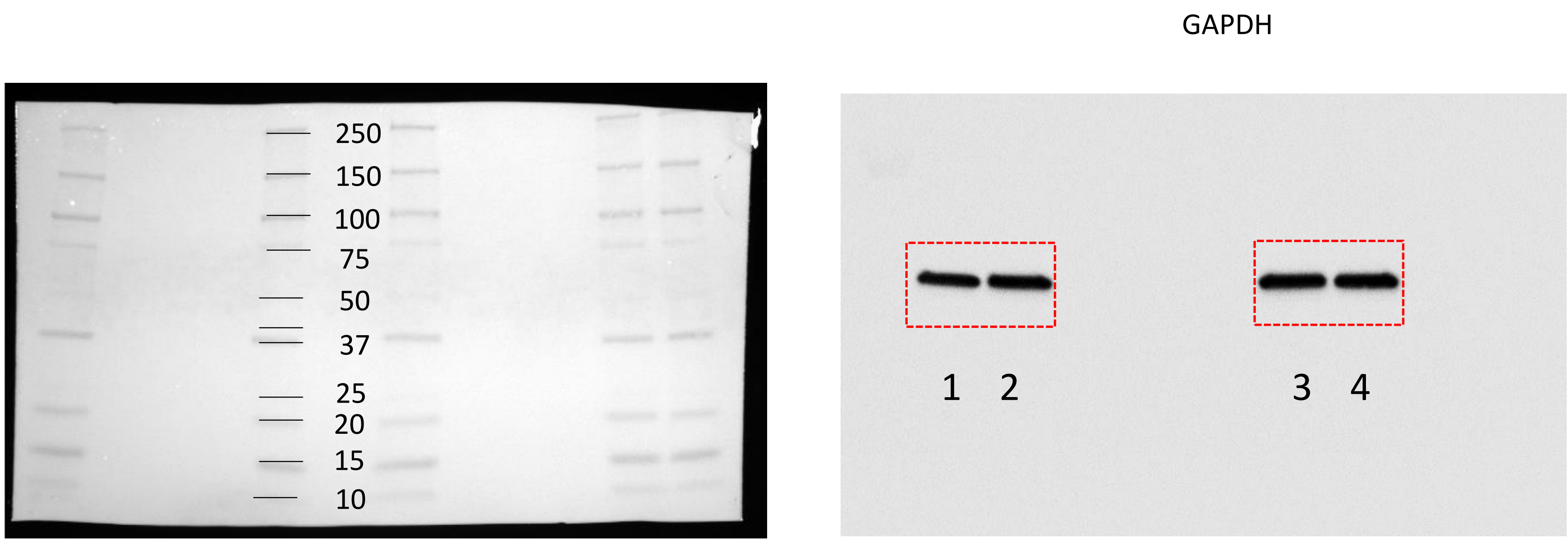

1 siNT, 72 h
2 siJPT1, 72 h
3 siNT, 168 h
4 siJPT1, 168 h

Supplemental Figure 4o.

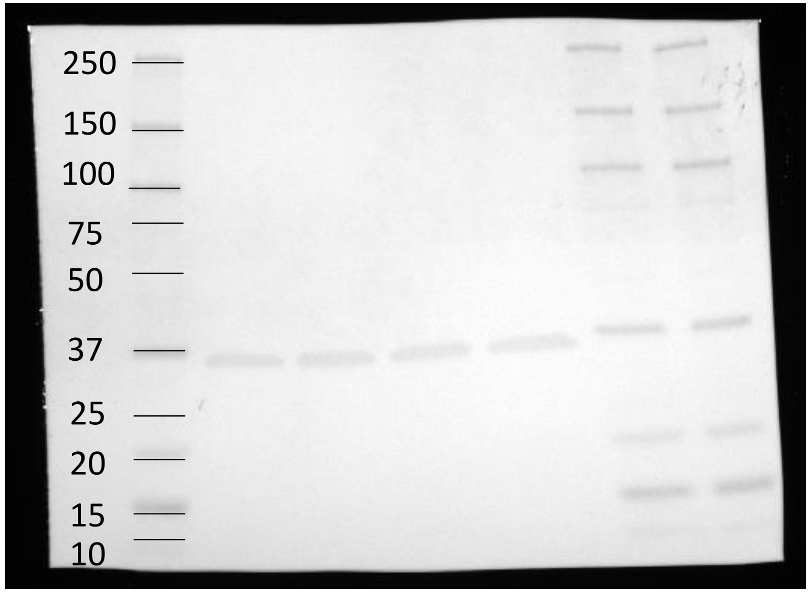

MYC

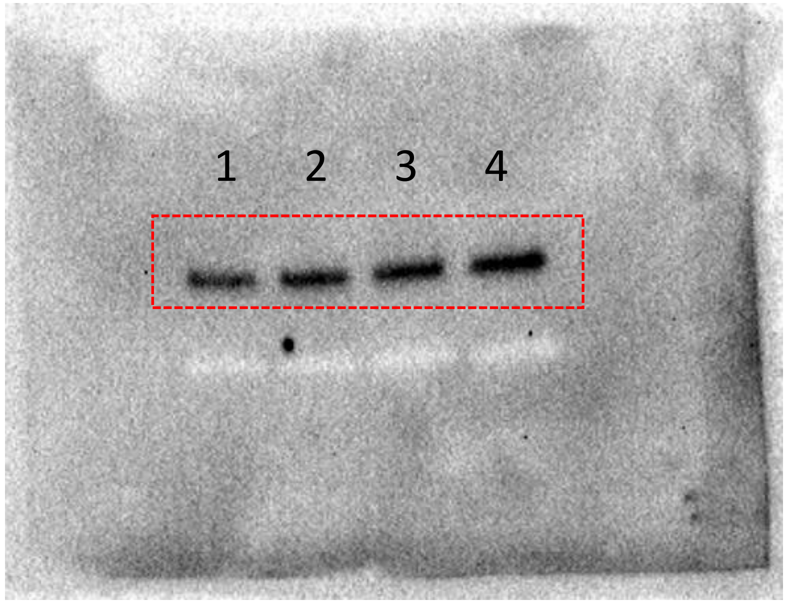

1 siNT, 72 h
2 siJPT1, 72 h
3 siNT, 168 h
4 siJPT1, 168 h

Supplemental Figure 4p.

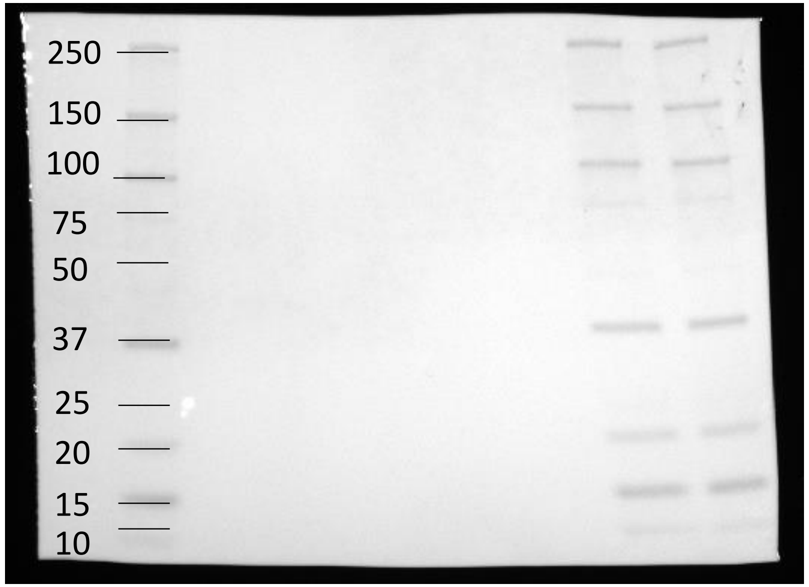

GAPDH

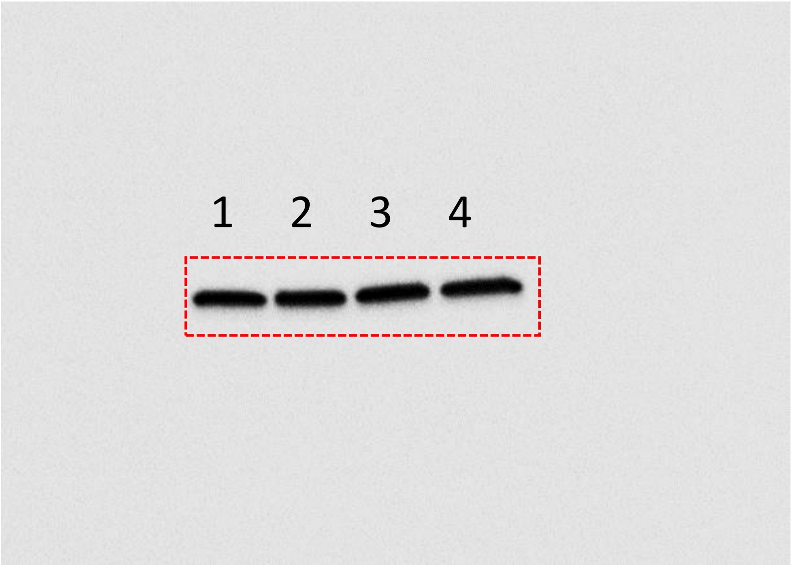

1 siNT, 72 h
2 siJPT1, 72 h
3 siNT, 168 h
4 siJPT1, 168 h

Supplemental Figure 4q.

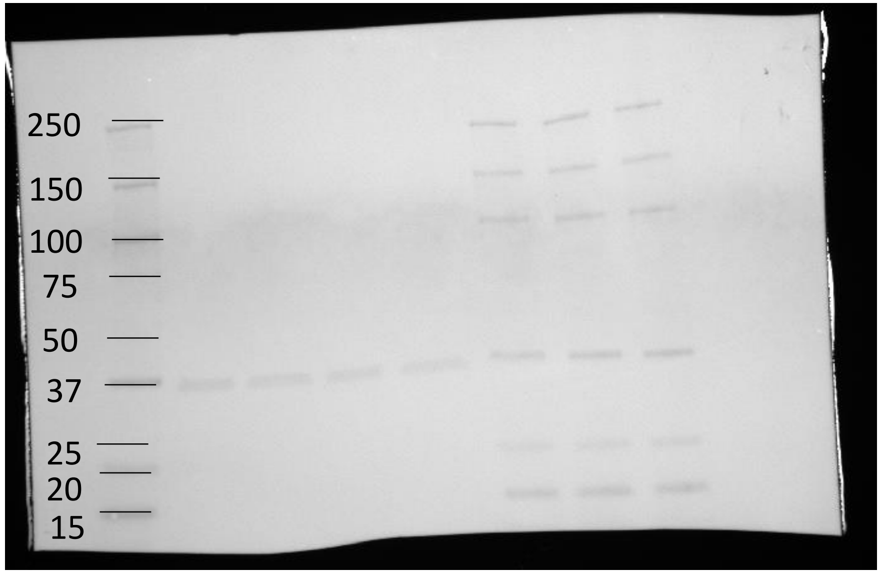

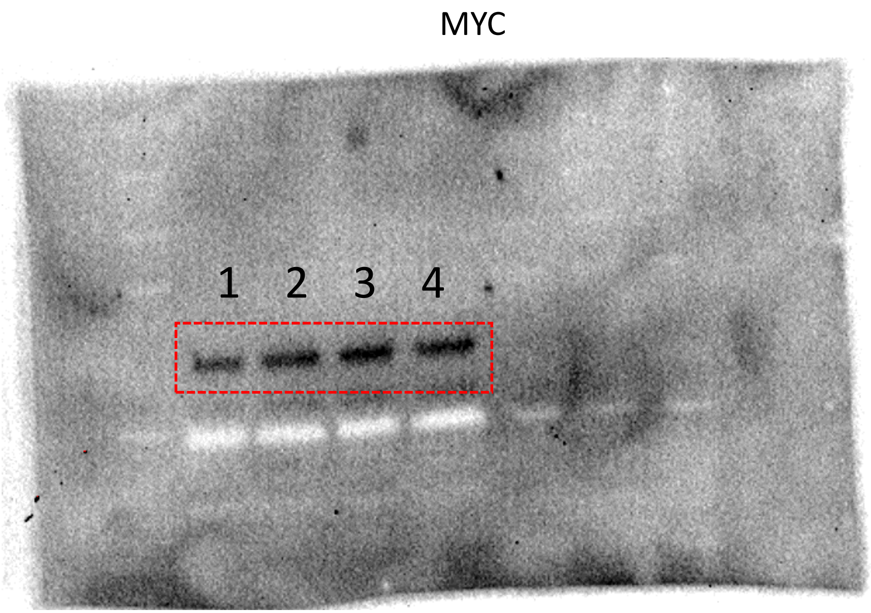

1 siNT, 72 h
2 siJPT1, 72 h
3 siNT, 168 h
4 siJPT1, 168 h

Supplemental Figure 4r.

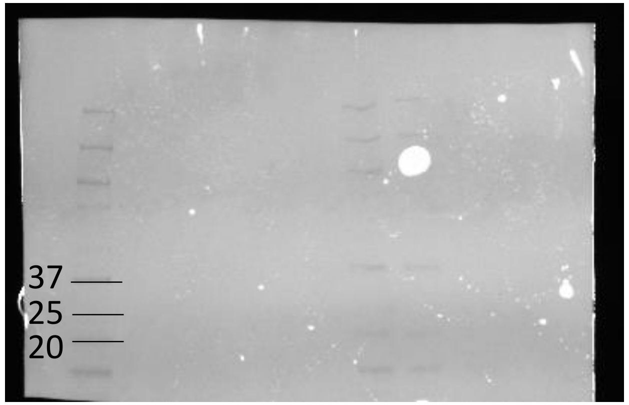

JPT1

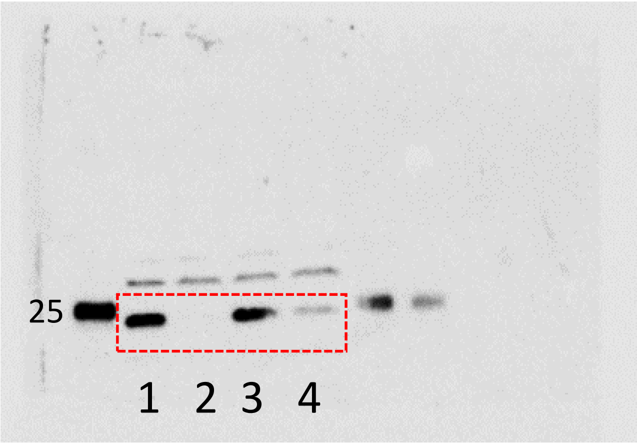

1 siNT, 72 h
2 siJPT1, 72 h
3 siNT, 168 h
4 siJPT1, 168 h

Supplemental Figure 4s.

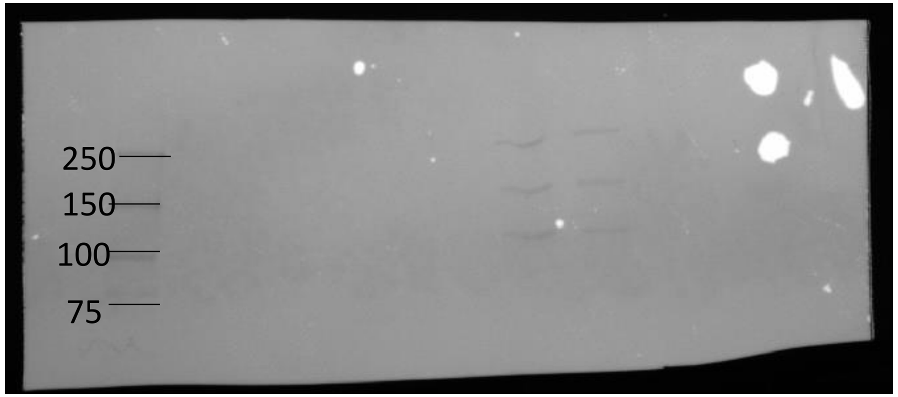

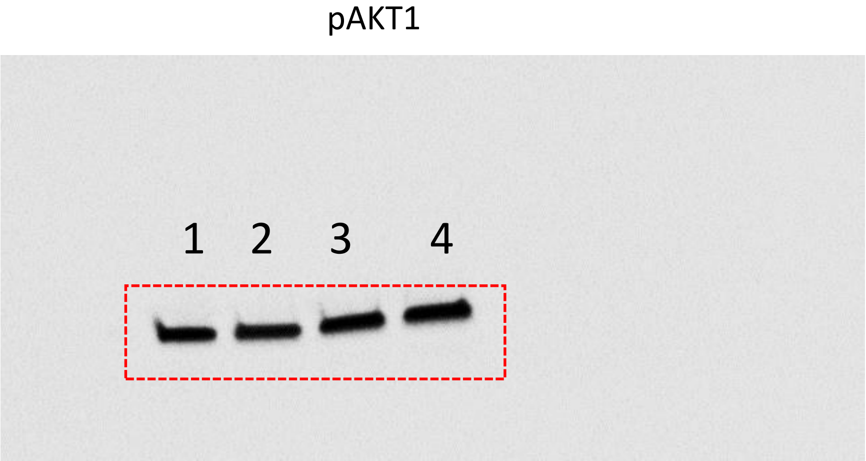

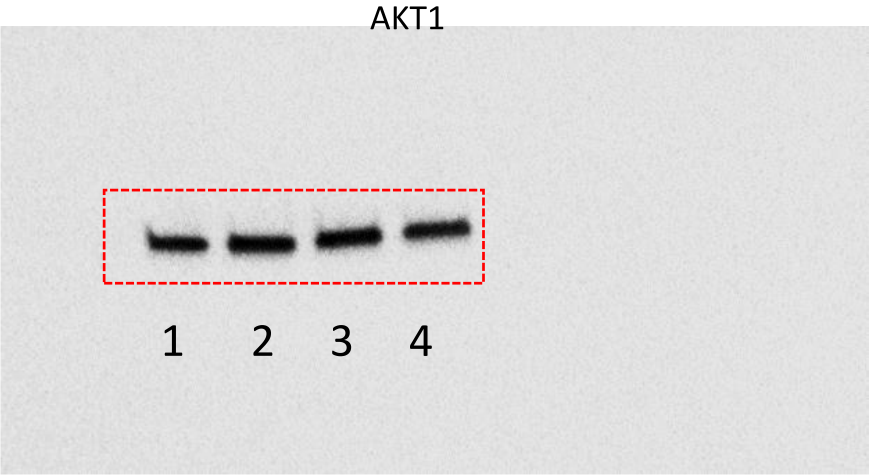

1 siNT, 72 h
2 siJPT1, 72 h
3 siNT, 168 h
4 siJPT1, 168 h

Supplemental Figure 4t.

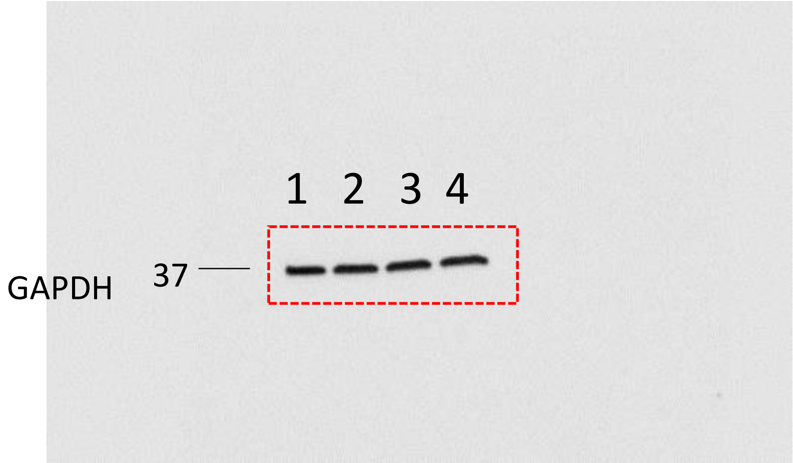

1 siNT, 72 h
2 siJPT1, 72 h
3 siNT, 168 h
4 siJPT1, 168 h

Supplemental Figure 4u.

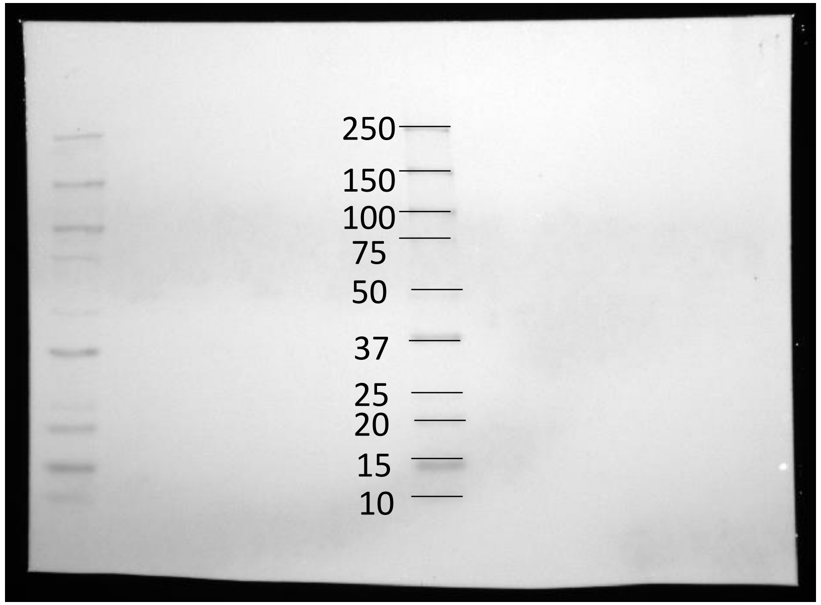

JPT1

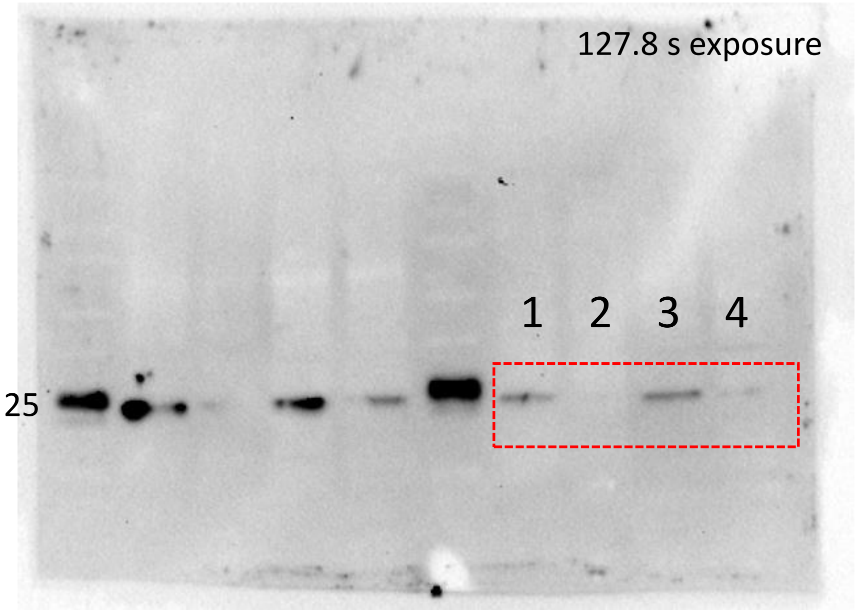

1 siNT, 72 h
2 siJPT1, 72 h
3 siNT, 168 h
4 siJPT1, 168 h

Supplemental Figure 4v.

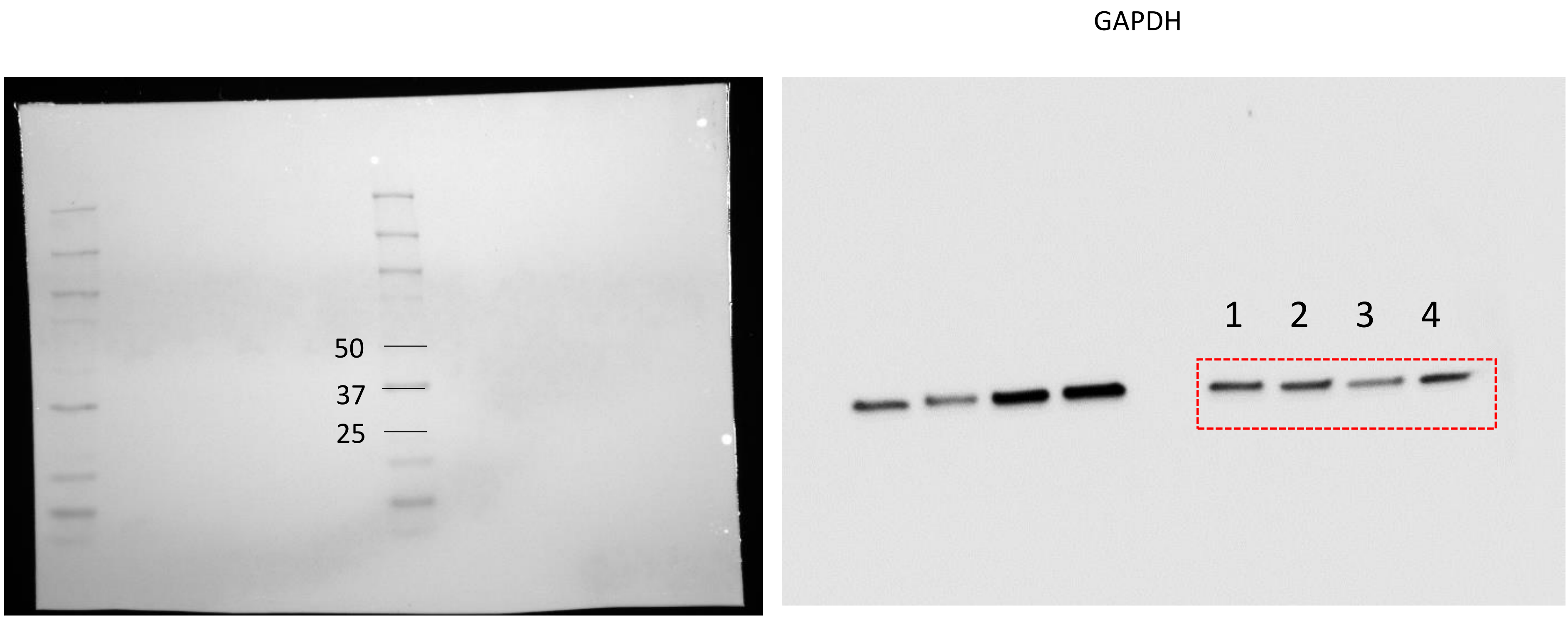

1 siNT, 72 h
2 siJPT1, 72 h
3 siNT, 168 h
4 siJPT1, 168 h

Supplemental Figure 4w.

JPT1

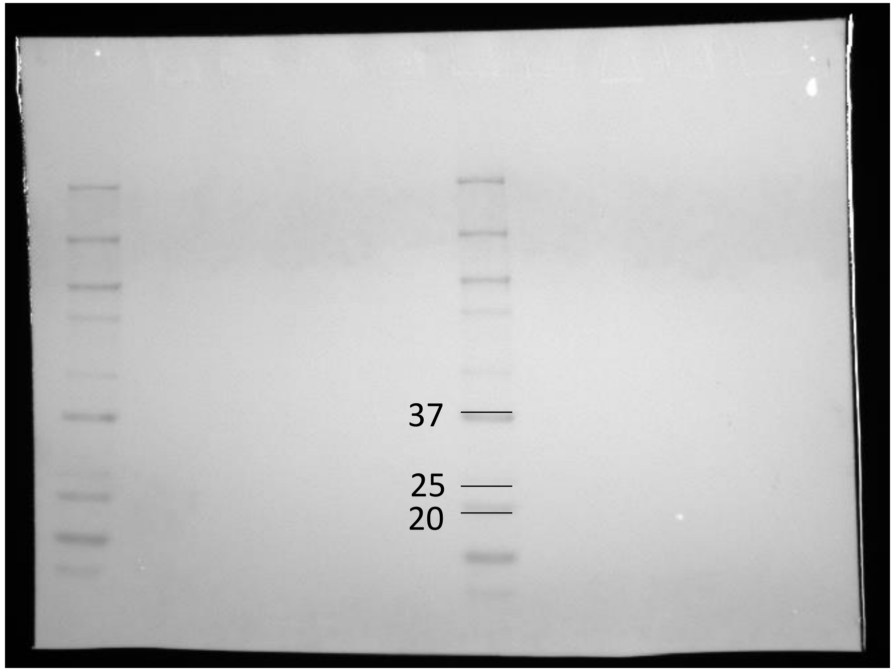

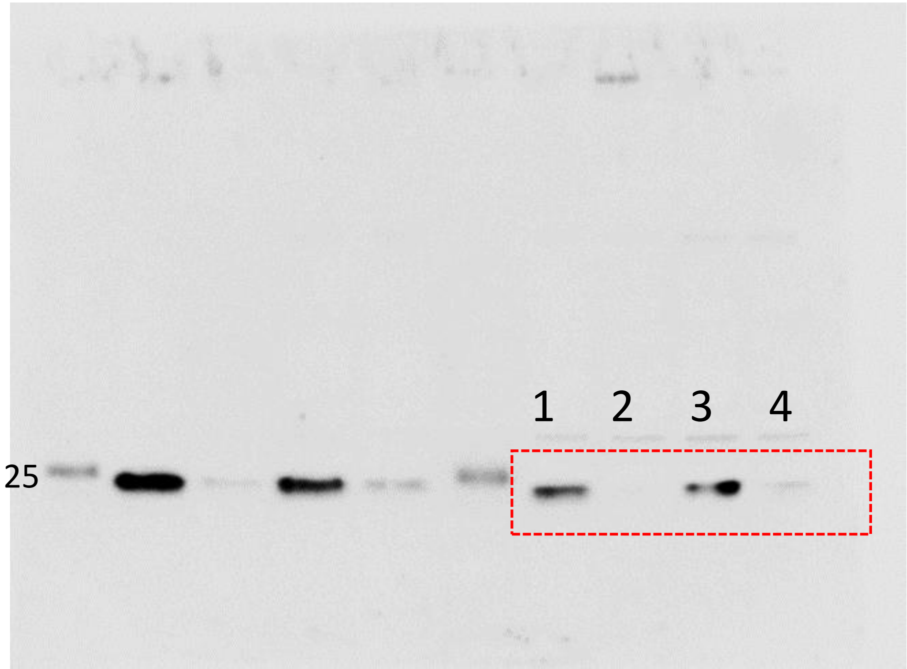

1 siNT, 72 h
2 siJPT1, 72 h
3 siNT, 168 h
4 siJPT1, 168 h

Supplemental Figure 4x.

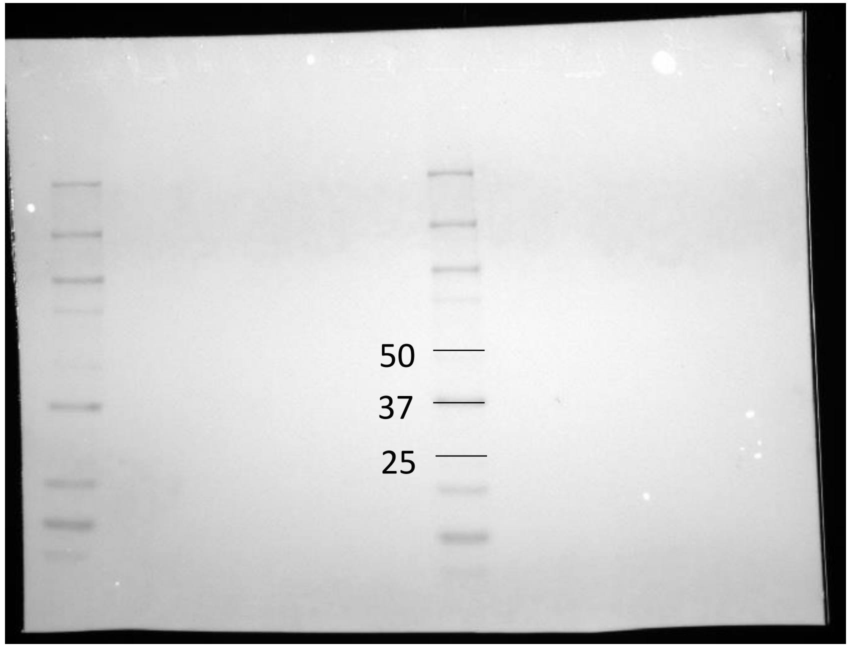

GAPDH

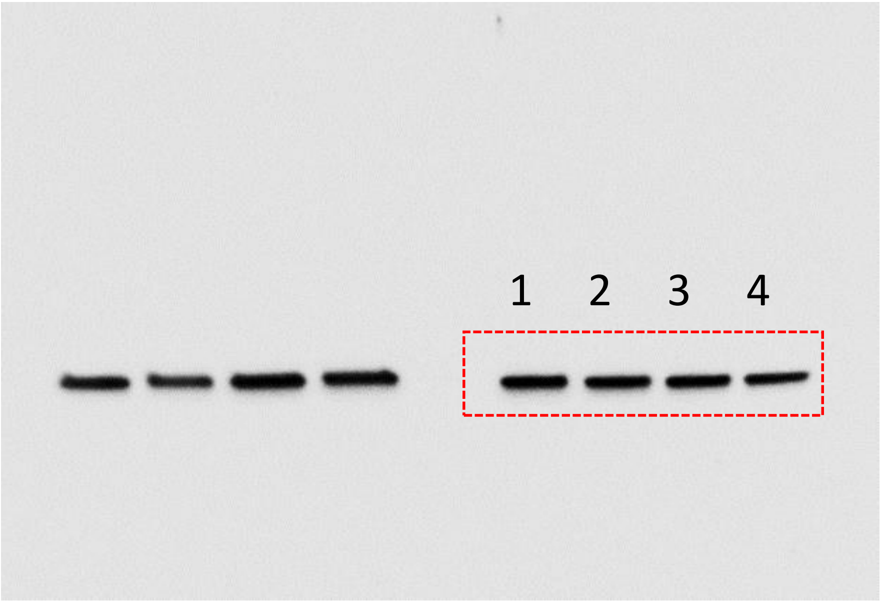

1 siNT, 72 h
2 siJPT1, 72 h
3 siNT, 168 h
4 siJPT1, 168 h

Supplemental Figure 4y.

Supp blot:
Set 1 proliferation (day 3 and day 7 WB)
ACI-181

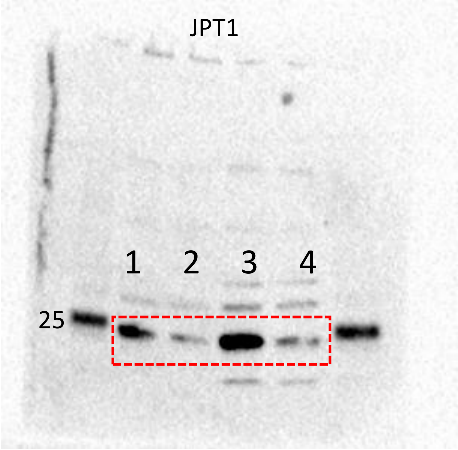

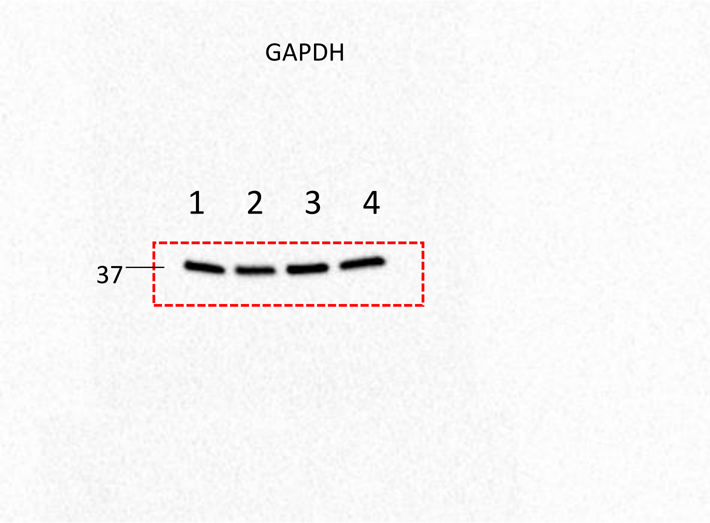

1 siNT, 72 h
2 siJPT1, 72 h
3 siNT, 168 h
4 siJPT1, 168 h

Supplemental Figure 4z.

Supp blot:
Set 1 proliferation (day 3 and day 7 WB)
RL-95

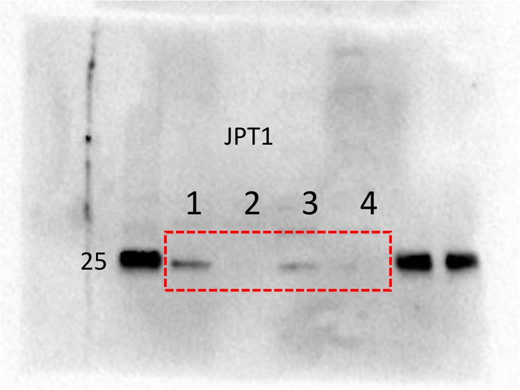

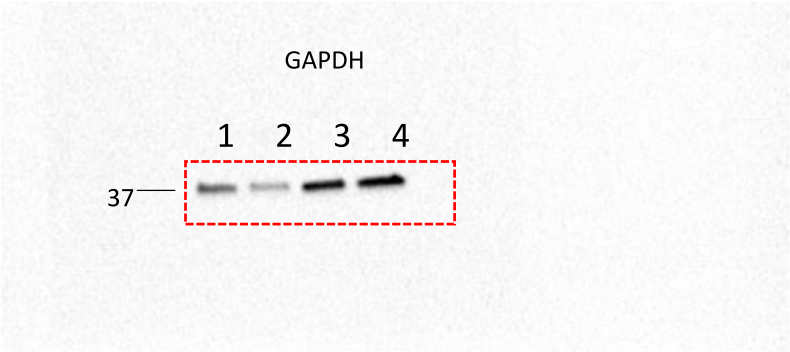

1 siNT, 72 h
2 siJPT1, 72 h
3 siNT, 168 h
4 siJPT1, 168 h

Supplemental Figure 4aa.

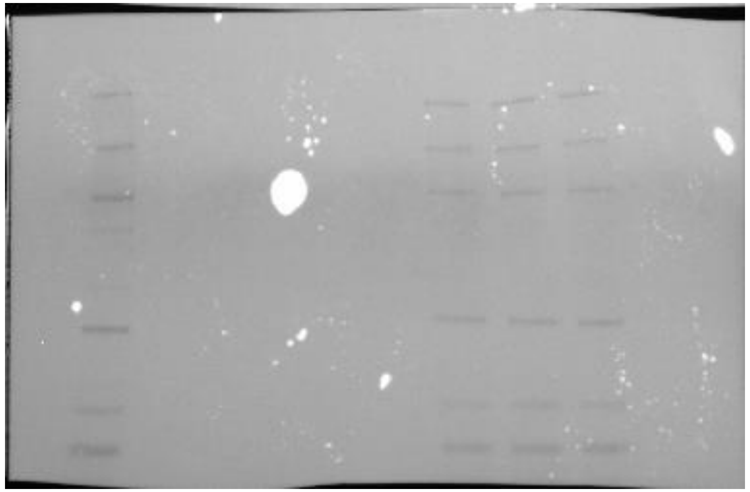

GAPDH

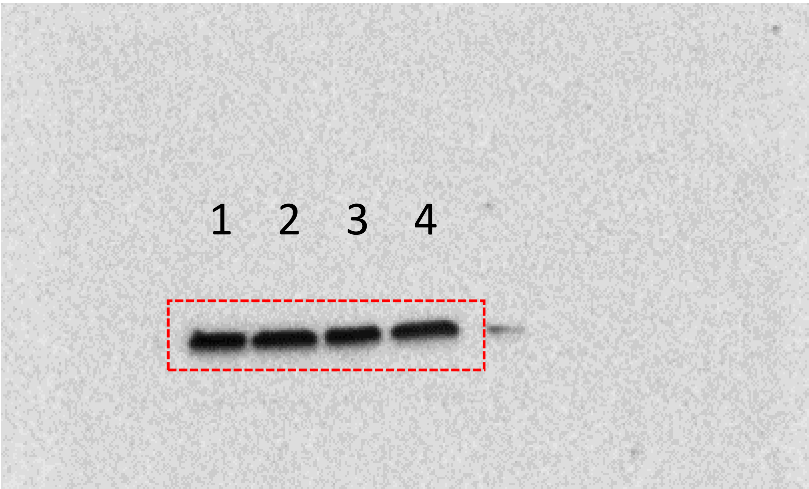

1 siNT, 72 h
2 siJPT1, 72 h
3 siNT, 168 h
4 siJPT1, 168 h
