# Supplementary material for: Jupiter microtubule‐associated homolog 1 (JPT1): A predictive and pharmacodynamic biomarker of metformin response in endometrial cancers
Source: Cancer Med. 2019 Dec 6;9(3):1092–103. doi: 10.1002/cam4.2729 (PMC6997075; doi:10.1002/cam4.2729)
Supplement: Supplementary file 5 [file CAM4-9-1092-s005.pdf]

Supplemental Figure 5.

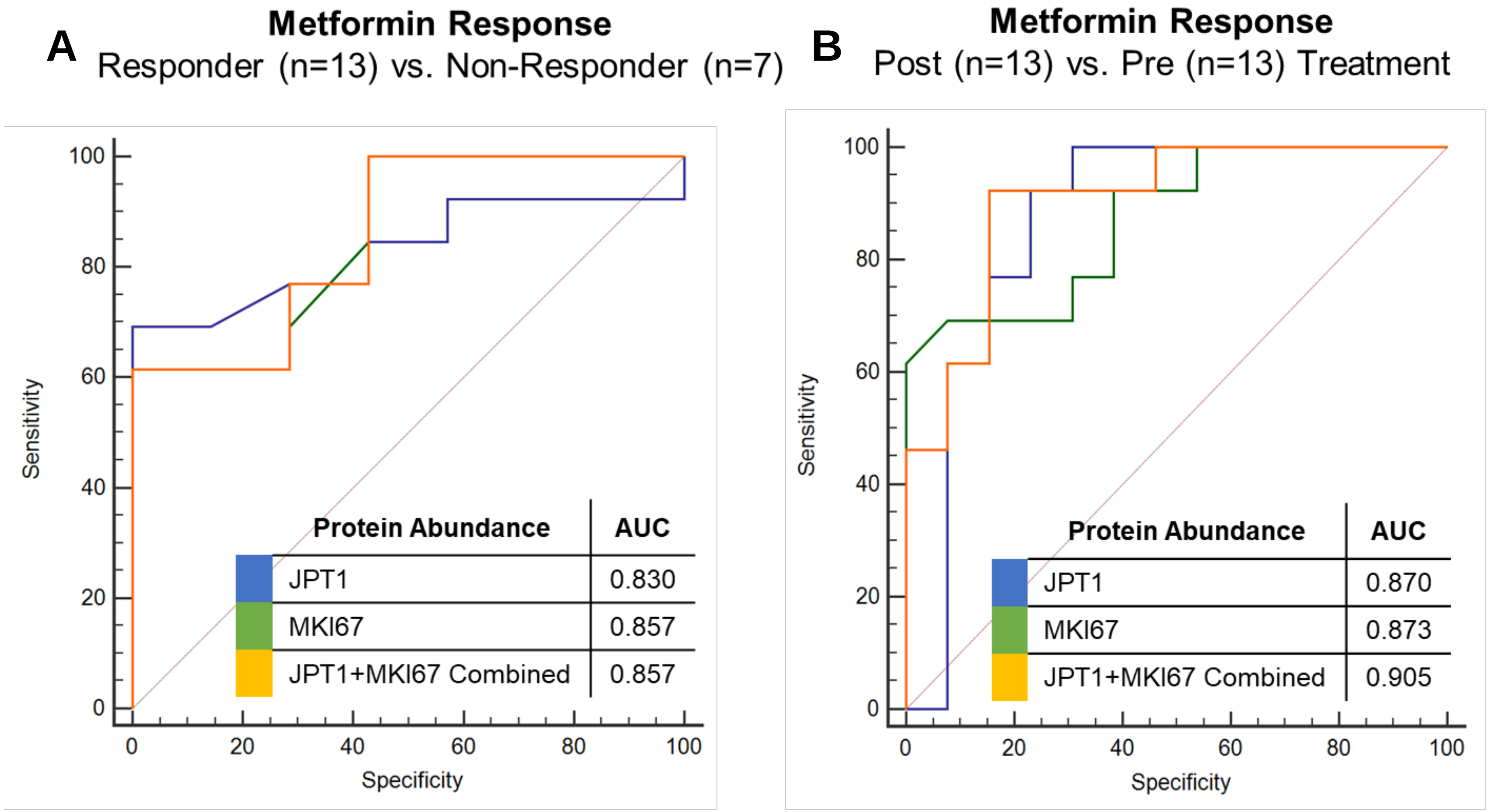

Protein Abundance	P-value (Resp vs Non-Resp)	P-value (Post vs Pre-Treatment)
JPT1 vs MKI67	0.783	0.9735
JPT1 vs Combined	0.7733	0.4585
MKI67 vs Combined	1.0	0.5181
