# Supplementary material for: Jupiter microtubule‐associated homolog 1 (JPT1): A predictive and pharmacodynamic biomarker of metformin response in endometrial cancers
Source: Cancer Med. 2019 Dec 6;9(3):1092–103. doi: 10.1002/cam4.2729 (PMC6997075; doi:10.1002/cam4.2729)
Supplement: Supplementary file 6 [file CAM4-9-1092-s006.pdf]

Supplemental Figure 6.

A Correlation of JPT1 and MKI67 Transcript Abundance in n=540 TCGA UCEC patients

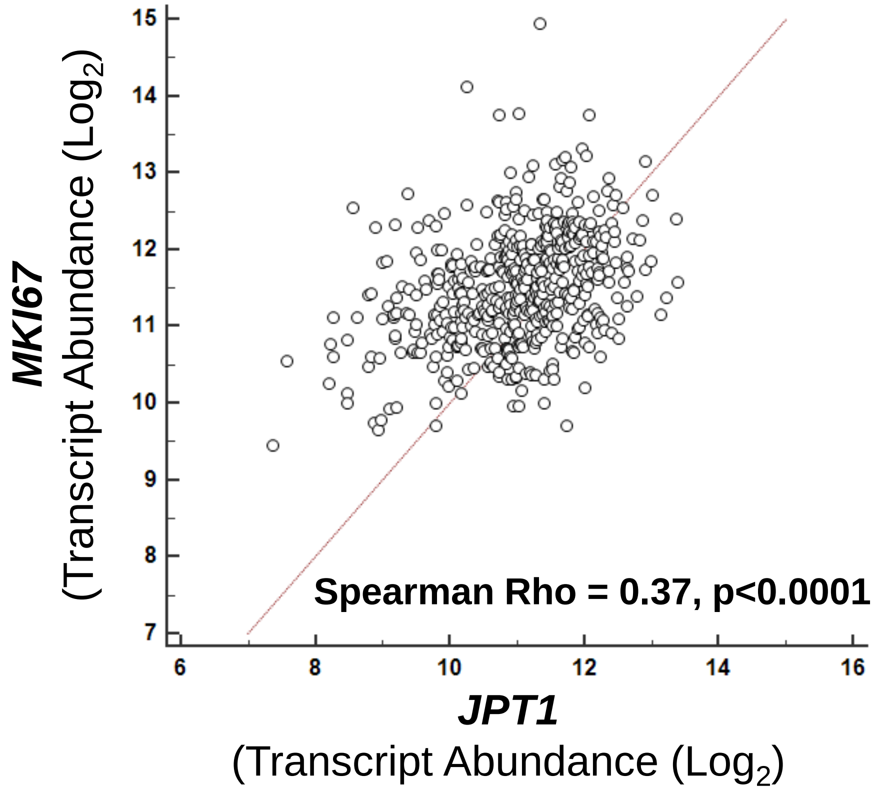

B Comparison of JPT1 Transcript Abundance and Overall Survival in n=540 TCGA UCEC patients

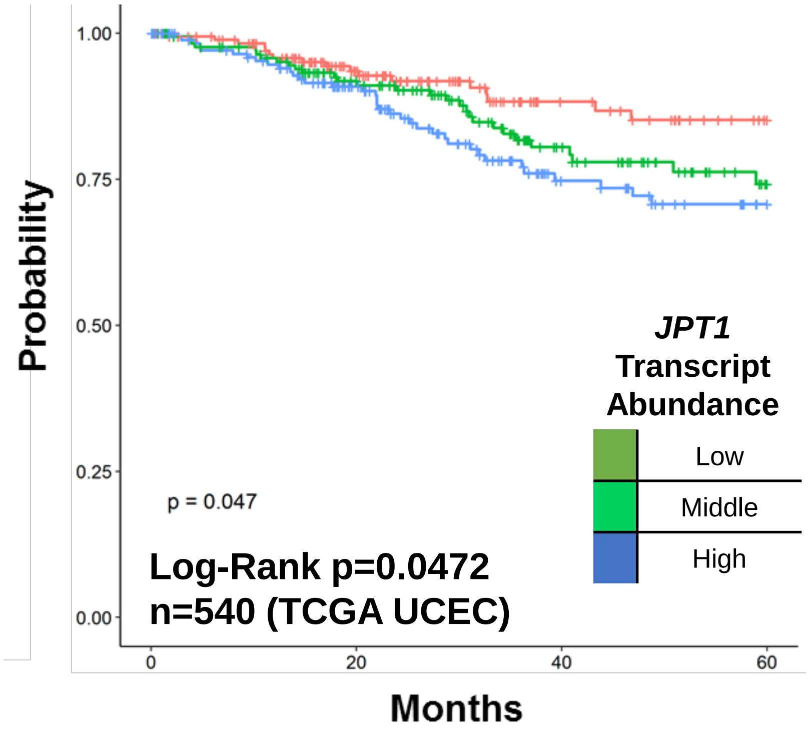

Hazard Ratio (High vs Low)	95% CI	Univariate Cox p-value
2.0456	1.148-3.641	0.0150
